# Data-driven models of dominantly-inherited Alzheimer’s disease progression

**DOI:** 10.1093/brain/awy050

**Published:** 2018-03-22

**Authors:** Neil P Oxtoby, Alexandra L Young, David M Cash, Tammie L S Benzinger, Anne M Fagan, John C Morris, Randall J Bateman, Nick C Fox, Jonathan M Schott, Daniel C Alexander

**Affiliations:** 1 Progression of Neurodegenerative Disease Group, Centre for Medical Image Computing, Department of Computer Science, University College London, Gower Street, London WC1E 6BT, UK; 2 Dementia Research Centre, Department of Neurodegenerative Disease, UCL Institute of Neurology, University College London, 8-11 Queen Square, London WC1N 3AR, UK; 3 Translational Imaging Group, Centre for Medical Image Computing, Department of Medical Physics and Biomedical Engineering, University College London, Gower Street, London WC1E 6BT, UK; 4 Department of Neurology, Washington University School of Medicine, St Louis, MO, 63110, USA; 5 UK Dementia Research Institute, University College London, London, UK

**Keywords:** event-based model, differential-equation model, disease progression, dominantly-inherited Alzheimer’s disease, biomarker dynamics

## Abstract

See Li and Donohue (doi:10.1093/brain/awy089) for a scientific commentary on this article.

Dominantly-inherited Alzheimer’s disease is widely hoped to hold the key to developing interventions for sporadic late onset Alzheimer’s disease. We use emerging techniques in generative data-driven disease progression modelling to characterize dominantly-inherited Alzheimer’s disease progression with unprecedented resolution, and without relying upon familial estimates of years until symptom onset. We retrospectively analysed biomarker data from the sixth data freeze of the Dominantly Inherited Alzheimer Network observational study, including measures of amyloid proteins and neurofibrillary tangles in the brain, regional brain volumes and cortical thicknesses, brain glucose hypometabolism, and cognitive performance from the Mini-Mental State Examination (all adjusted for age, years of education, sex, and head size, as appropriate). Data included 338 participants with known mutation status (211 mutation carriers in three subtypes: 163 *PSEN1*, 17 *PSEN2*, and 31 *APP*) and a baseline visit (age 19–66; up to four visits each, 1.1 ± 1.9 years in duration; spanning 30 years before, to 21 years after, parental age of symptom onset). We used an event-based model to estimate sequences of biomarker changes from baseline data across disease subtypes (mutation groups), and a differential equation model to estimate biomarker trajectories from longitudinal data (up to 66 mutation carriers, all subtypes combined). The two models concur that biomarker abnormality proceeds as follows: amyloid deposition in cortical then subcortical regions (∼24 ± 11 years before onset); phosphorylated tau (17 ± 8 years), tau and amyloid-β changes in cerebrospinal fluid; neurodegeneration first in the putamen and nucleus accumbens (up to 6 ± 2 years); then cognitive decline (7 ± 6 years), cerebral hypometabolism (4 ± 4 years), and further regional neurodegeneration. Our models predicted symptom onset more accurately than predictions that used familial estimates: root mean squared error of 1.35 years versus 5.54 years. The models reveal hidden detail on dominantly-inherited Alzheimer’s disease progression, as well as providing data-driven systems for fine-grained patient staging and prediction of symptom onset with great potential utility in clinical trials.

## Teaser

The key to developing interventions for Alzheimer’s disease may lie within the less common familial form, which has a predictable presymptomatic phase. Oxtoby *et al.* use modern computational techniques to characterize familial Alzheimer’s disease progression. The resulting data-driven models allow fine-grained patient staging, with potential utility in clinical trials.

## Introduction

Understanding and identifying the earliest pathological changes of Alzheimer’s disease is key to realizing disease-modifying treatments, which are likely to be most efficacious when given early. However, identifying individuals in the presymptomatic stage of typical, sporadic, late onset Alzheimer’s disease is challenging. Therefore, there is considerable interest in investigating dominantly-inherited Alzheimer’s disease, which is caused by mutations in the amyloid precursor protein (*APP*), presenilin 1 (*PSEN1*), and presenilin 2 (*PSEN2*) genes, and which provides the opportunity to identify asymptomatic ‘at risk’ individuals prior to the onset of cognitive decline for observational studies and clinical trials. Although considerably rarer than sporadic Alzheimer’s disease, dominantly-inherited Alzheimer’s disease has broadly similar clinical presentation (Ryan *et al.*, 2016; Tang *et al.*, 2016), i.e. episodic memory followed by further cognitive deficits, and both display heterogeneity in terms of symptoms and progression, much of which is unexplained (Bateman *et al.*, 2011). An important question when attempting to extrapolate biomarker dynamics (and in due course clinical trials results) between dominantly-inherited Alzheimer’s disease and sporadic Alzheimer’s disease, is whether presymptomatic changes in dominantly-inherited Alzheimer’s disease mirror those in sporadic Alzheimer’s disease, as might be expected given the broad similarities in pathological features across both diseases (Bateman *et al.*, 2011; Morris *et al.*, 2012; Weiner *et al.*, 2012; Cairns *et al.*, 2015).

Most previous investigations into dominantly-inherited Alzheimer’s disease progression used traditional regression models to explore the time course of Alzheimer’s disease markers as a function of familial estimates of years to onset of clinical symptoms, based on age of onset (Ryman *et al.*, 2014) in affected first-degree relatives. In 2012, this type of cross-sectional analysis of biomarker trajectories in the Dominantly Inherited Alzheimer Network (DIAN) observational study estimated the following sequence of presymptomatic biomarker changes (Bateman *et al.*, 2012): measures of amyloid-β in CSF and in standardized uptake value ratio (SUVR) from amyloid imaging using Pittsburgh compound B PET (PiB-PET); CSF levels of tau; regional brain atrophy; SUVR for cortical glucose hypometabolism in fluorodeoxyglucose PET (FDG-PET); episodic memory; Mini-Mental State Examination (MMSE) score (Folstein *et al.*, 1975); and Clinical Dementia Rating (CDR) Sum of Boxes score (Berg, 1988). Results of a more recent model-based analysis (Fleisher *et al.*, 2015) showed a similar progression sequence in the Alzheimer’s Prevention Initiative Colombian cohort, all of whom carry the same mutation (E280A PSEN1). A more detailed investigation of imaging biomarkers (Benzinger *et al.*, 2013) observed regional variability in the cross-sectional sequence of biomarker changes: some grey matter structures having amyloid plaques may not later lose metabolic function, and others may not atrophy. Various other studies of dominantly-inherited Alzheimer’s disease have reported early behavioural changes (Ringman *et al.*, 2015) and presymptomatic within-individual atrophy (Cash *et al.*, 2013) in brain regions commonly associated with sporadic Alzheimer’s disease, and additionally in the putamen and thalamus. The key feature in each of these studies of dominantly-inherited Alzheimer’s disease progression is the reliance upon familial estimates of years to onset, which is typically based upon the estimated age at which an individual’s affected parent first shows progressive cognitive decline (Bateman *et al.*, 2011), or upon the average age of onset for a mutation type (Ryman *et al.*, 2014). The parental estimate of familial age of onset is generated by a semi-structured interview and is known to be inherently uncertain both because of uncertainties in estimating when an individual is deemed to be affected, and because there can be substantial within-family and within-mutation differences in actual age of onset (Ryman *et al.*, 2014). This uncertainty in familial age of onset limits its utility for estimating disease progression in presymptomatic individuals who carry a dominantly-inherited Alzheimer’s disease mutation: reducing confidence in predicting onset; and when staging patients—at best reducing the resolution in which biomarker ordering can be inferred, at worst biasing the ordering.

Here we take a different approach: generative, data-driven, disease progression modelling. Data-driven progression models have emerged in recent years as a family of computational approaches for analysing progressive diseases. Instead of regressing against predefined disease stages (Scahill *et al.*, 2002; Ridha *et al.*, 2006; Yang *et al.*, 2011; Bateman *et al.*, 2012), or learning to classify cases from a labelled training database (Klöppel *et al.*, 2008; Mattila *et al.*, 2011; Young *et al.*, 2013), generative data-driven progression models construct an explicit quantitative disease signature without the need for *a priori* staging. Mostly applied to neurodegenerative conditions like Alzheimer’s disease, results include discrete models of biomarker changes (Fonteijn *et al.*, 2012; Young *et al.*, 2014; Venkatraghavan *et al.*, 2017), continuous models of biomarker dynamics (Jedynak *et al.*, 2012; Villemagne *et al.*, 2013; Donohue *et al.*, 2014; Oxtoby *et al.*, 2014), spatiotemporal models of brain image dynamics (Durrleman *et al.*, 2013; Lorenzi *et al.*, 2015; Schiratti *et al.*, 2015; Huizinga *et al.*, 2016), and models of disease propagation mechanisms (Seeley *et al.*, 2009; Raj *et al.*, 2012; Zhou *et al.*, 2012; Iturria-Medina *et al.*, 2014, 2017). For a recent review of the field of data-driven disease progression modelling, see Oxtoby *et al.* (2017).

In this study we use two generative data-driven disease progression models to extract patterns of observable biomarker changes in dominantly-inherited Alzheimer’s disease. We estimate ordered sequences of biomarker abnormality in disease subtypes (mutation groups) from cross-sectional data using an event-based model (Fonteijn *et al.*, 2012; Young *et al.*, 2014), and we estimate long-term biomarker trajectories from short-interval longitudinal data using a non-parametric differential equation model similar to previous parametric work (Villemagne *et al.*, 2013; Oxtoby *et al.*, 2014). Our data-driven generative models have several potential advantages over previous models. First, they are generalizable to non-familial forms of progressive diseases because they do not rely on familial age of onset. Second, they generate a uniquely detailed sequence of biomarker changes and trajectories. Third, they support a fine-grained staging system of potential direct application to clinical trials and clinical practice. We demonstrate the prognostic utility by predicting actual symptom onset in unseen data more accurately than using estimates based on familial age of onset.

## Materials and methods

We used data-driven models to analyse biomarker data (MRI, PET, CSF, cognitive test scores) from the DIAN study. From cross-sectional (baseline) data we estimated disease progression sequences using an event-based model (Fonteijn *et al.*, 2012; Young *et al.*, 2014). For explicit quantification of disease progression time, we estimated long-term biomarker trajectories from short-term longitudinal data by using covariate-adjusted, non-parametric differential equation models, which offer two key advantages over previous approaches in (Villemagne *et al.*, 2013; Oxtoby *et al.*, 2014): replacing parametric model selection with a data-driven approach, and explicitly estimating population variance in a Bayesian manner. See ‘Statistical analysis’ section for more details.

### Participants

At the sixth data freeze, the DIAN cohort included 338 individual participants (192 females, 57%) with known mutation status and a baseline visit, aged 19–66 years at baseline (39 ± 10 years), with up to four visits each (1.1 ± 1.9 years in duration, total of 535 visits), spanning 30 years before and 21 years after parental age of symptom onset. For detailed descriptive summaries of the cohort, we refer the reader to Morris *et al.* (2012).

### Data selection and preparation

Table 1 summarizes the demographics of DIAN participants analysed in this work.

**Table 1:** Demographics for DIAN participants at Data Freeze 6

Demographic	Non-carriers	Mutation carriers, *n* [PSEN1, PSEN2, APP (%)]
**Cross-sectional (event-based models), *n* analysed**	127	211 [163, 17, 31 (77, 8, 15)]
	Cog: 121	Cog: 194 [150, 15, 29 (77, 8, 15)]
	MRI: 104	MRI: 159 [124, 11, 24 (78, 7, 15)]
	CSF: 94	CSF: 162 [126, 14, 22 (78, 9, 13)]
	PiB: 98	PiB: 139 [107, 11, 21 (77, 8, 15)]
	FDG: 98	FDG: 148 [113, 11, 24 (76, 8, 16)]
Female, *n* (%)	75 (59%)	117 (55) [92, 5, 20 (79, 4, 17)]
*APOE* ɛ4-positive	37 (29%)	61 (29) [47, 7, 7 (77, 11.5, 11.5)]
	Cog: 35	Cog: 59 [45, 7, 7 (76, 12, 12)]
	MRI: 29	MRI: 46 [34, 5, 7 (74, 11, 15)]
	CSF: 31	CSF: 50 [37, 7, 6 (74, 14, 12)]
	PiB: 29	PiB: 42 [30, 5, 7 (71, 12, 17)]
	FDG: 27	FDG: 44 [32, 5, 7 (73, 11, 16)]
*APOE* ɛ4-negative	90 (71%)	150 (71) [116, 10, 24 (77, 7, 16)]
	Cog: 86	Cog: 135 [105, 8, 22 (78, 6, 16)]
	MRI: 75	MRI: 113 [90, 6, 17 (80, 5, 15)]
	CSF: 64	CSF: 112 [89, 7, 16 (80, 6, 14)]
	PiB: 69	PiB: 97 [77, 6, 14 (79, 6, 15)]
	FDG: 71	FDG: 104 [81, 6, 17 (78, 6, 16)]
Age at baseline ± SD, years	39 ± 10	39 ± 10 [39 ± 10, 39 ± 10, 43 ± 10]
Education at baseline ± SD, years	15 ± 3	14 ± 3 [14 ± 3, 15 ± 3, 14 ± 3]
EYO at baseline ± SD, years	−7 ± 12	−7 ± 10 [−7 ± 10, −12 ± 10, −6 ± 9]
**Longitudinal (differential equation models) *n* analysed**	n/a	Cog: 51 [41, 1, 9 (80, 2, 18)]
		MRI: 46 [36, 2, 8 (78, 4.5, 17.5)]
		CSF: 31 [27, 1, 3 (87, 3, 10)]
		PiB: 30 [23, 2, 5 (77, 7, 16)]
		FDG: 38 [30, 2, 6 (79, 5, 16)]
Female	n/a	Cog: 28 (55) [21, 1, 6 (75, 4, 21)]
		MRI: 26 (56) [19, 1, 6 (73, 4, 23)]
		CSF: 16 (52) [13, 0, 3 (81, 0, 19)]
		PiB: 16 (53) [11, 1, 4 (69, 6, 25)]
		FDG: 22 (58) [16, 1, 5 (73, 5, 23)]
*APOE* ɛ4-positive	n/a	Cog: 17 (33) [13, 0, 4 (76, 0, 24)]
		MRI: 16 (35) [11, 1, 4 (69, 6, 25)]
		CSF: 8 (26) [6, 1, 1 (75, 12.5, 12.5)]
		PiB: 13 (43) [9, 1, 3 (69, 8, 23)]
		FDG: 14 (37) [10, 1, 3 (71, 7, 21)]
*APOE* ɛ4-negative	n/a	Cog: 34 (67) [28, 1, 5 (82, 3, 15)]
		MRI: 30 (65) [25, 1, 4 (84, 3, 13)]
		CSF: 23 (74) [21, 0, 2 (91, 0, 9)]
		PiB: 17 (57) [14, 1, 2 (82, 6, 12)]
		FDG: 24 (63) [20, 1, 3 (83, 4, 13)]
Age at baseline ± SD, years	n/a	Cog: 41 ± 10 [40 ± 10, 32 ± 0, 48 ± 7]
		MRI: 42 ± 10 [40 ± 10, 45 ± 18, 50 ± 6]
		CSF: 43 ± 9 [41 ± 9, 57 ± 0, 48 ± 8]
		PiB: 42 ± 10 [41 ± 10, 45 ± 18, 49 ± 4]
		FDG: 42 ± 10 [41 ± 10, 45 ± 18, 48 ± 5]
Education at baseline ± SD, years	n/a	Cog: 14 ± 2 [14 ± 2, 18 ± 0, 15 ± 2]
		MRI: 14 ± 2 [14 ± 2, 15 ± 4, 15 ± 2]
		CSF: 14 ± 3 [14 ± 3, 12 ± 0, 14 ± 3]
		PiB: 14 ± 3 [14 ± 2, 15 ± 4, 15 ± 3]
		FDG: 14 ± 2 [14 ± 2, 15 ± 4, 15 ± 2]
EYO at baseline ± SD, years	n/a	Cog: −3 ± 7 [−3 ± 7, −19 ± 0, −2 ± 8]
		MRI: −3 ± 7 [−3 ± 7, −6 ± 18, 0 ± 6]
		CSF: −1 ± 7 [−1 ± 7, 7 ± 0, −3 ± 7]
		PiB: −3 ± 6 [−3 ± 6, −6 ± 18, 0 ± 3]
		FDG: −4 ± 7 [−4 ± 7, −6 ± 18, −2 ± 5]

*Top*: Cross-sectional data used to build event-based models of dominantly-inherited Alzheimer’s disease progression.

*Bottom*: Longitudinal data used to build differential equation models of dominantly-inherited Alzheimer’s disease progression. See main text for further details. Percentages given to within 1%.

Cog = cognitive test scores; EYO = estimated years to onset based on parental age of symptom onset; FDG = fludeoxyglucose hypometabolism PET data; SD = standard deviation.

**Figure 1:**
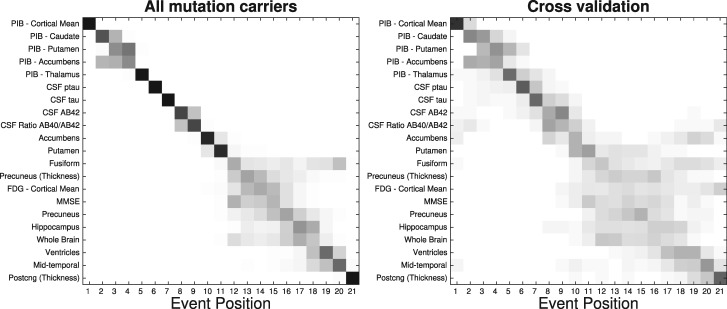
**Event-based model of dominantly-inherited Alzheimer’s disease progression.** Positional variance diagrams. *Left*: Event-based model estimated on all mutation carriers in the DIAN dataset. *Right*: Cross-validation through bootstrapping. The vertical ordering (*top* to *bottom*) is given by the maximum likelihood sequence estimated by the model. Greyscale intensity represents posterior confidence in each event’s position (each row), from Markov chain Monte Carlo samples of the posterior (*left*) or from bootstrapping (*right*). AB = amyloid-β; Postcng = posterior cingulate; ptau = phosphorylated tau.

We selected 24 Alzheimer’s disease biomarkers based on specificity to the disease, or if disease ‘signal’ is present, i.e. quantifiable distinction between mutation carriers and non-carriers (see ‘Statistical analysis’ section). The biomarkers include CSF measures of molecular pathology (amyloid proteins and neurofibrillary tangles); a cognitive test score (MMSE); regional brain volumetry from MRI, e.g. hippocampus, middle-temporal region, temporo-parietal cortex; PiB-PET imaging SUVR measures of amyloid accumulation; and FDG-PET imaging SUVR measures of glucose hypometabolism. We excluded imaging data (21 structural scans from 10 participants) having artefacts or non-Alzheimer’s disease pathology such as a brain tumour. Of the included participants, 211 (117 females, 55%) were dominantly-inherited Alzheimer’s disease mutation carriers: 163 *PSEN1*, 17 *PSEN2*, and 31 *APP*; 120 were non-carriers. Baseline data for the mutation carriers and non-carriers was used to fit event-based models. The full set of biomarkers included in the event-based model is listed on the vertical axis of Fig. 1.

Of the 211 included mutation carriers, 66 had longitudinal data necessary for fitting differential equation models. To reduce the influence of undue measurement noise we excluded biomarker data with a large coefficient of variation within individuals, e.g. as done for CSF biomarkers in Bateman *et al.* (2012). Following Villemagne *et al.* (2013), we also excluded differential data that were both normal (beyond a threshold determined by clustering), and non-progressing (rate of change has a contradictory sign to disease progression, e.g. reverse atrophy or improved cognition). Finally, we identified six cognitively normal mutation carriers who developed symptoms during the study (global CDR becoming nonzero after baseline). Since we use our differential equation models to predict symptom onset for these participants, we excluded them from the model fits to avoid circularity (including them does not alter our results considerably). This left data from up to 51 mutation carriers (41 *PSEN1*, one *PSEN2*, nine *APP*; 28 females) available for analysis using differential equation models. Subsets had data for structural imaging (46; 26 females), CSF (31; 16 females), PiB PET (30; 16 females), and FDG PET (38; 22 females) biomarkers (Table 1). The number of data points included (and excluded) per biomarker were: MMSE *n = *51 (8); tau *n = *26 (9) and phosphorylated tau *n = *31 (4) in CSF; amyloid SUVR in the caudate *n = *28 (2), putamen *n = *29 (1), nucleus accumbens *n = *26 (4), and the cortical mean *n = *30 (0) from PiB-PET images; glucose hypometabolism SUVR in the posterior cingulate *n = *37 (1), hippocampus *n = *35 (3), and the cortical mean *n = *35 (3) from FDG-PET images; regional brain volumes from structural MRI in the nucleus accumbens *n = *40 (6), caudate *n = *41 (5), entorhinal area *n = *45 (1), fusiform gyrus *n = *42 (4), hippocampus *n = *44 (2), middle-temporal gyrus *n = *45 (1), precuneus *n = *44 (2), putamen *n = *42 (4), thalamus *n = *41 (5), ventricles *n = *44 (2), and whole brain *n = *43 (3); and average cortical thickness of the precuneus *n = *46 (0), posterior cingulate *n = *43 (3), entorhinal cortex *n = *44 (2), fusiform gyrus *n = *45 (1), and middle-temporal gyrus *n = *45 (1). All regional biomarkers in the brain are bilateral.

We used stepwise regression to remove the influence of age, years of education, sex, and head size (total intracranial volume, MRI volumes only) prior to fitting our models.

### Models

#### Cross-sectional: event-based models

The event-based model infers a sequence in which biomarkers show abnormality, together with uncertainty in that sequence, from cross-sectional data (Fonteijn *et al.*, 2012). This longitudinal picture of disease progression is estimable using this approach because, across the spectrum of DIAN study participants from cognitively normal controls (non-carriers of dominantly-inherited Alzheimer’s disease mutations), to presymptomatic mutation carriers, and symptomatic patients, more individuals will show higher likelihood of abnormality in biomarkers that change early in the progression. Thus, with sufficient representation across combinations of abnormal and normal observations, the likelihood of any full ordered sequence can be estimated to reveal the most likely sequences. The probabilistic sequence of events estimated by the event-based model is useful for fine-grained staging of individuals by calculating the likelihood of their data (biomarker observations) arising from each stage of the sequence (Young *et al.*, 2014).

We fit an event-based model to determine the most probable sequence of biomarker abnormality events and the uncertainty in this sequence for all but 3 of 24 biomarkers described previously: measurements of entorhinal cortex, thalamus, and caudate volume were excluded on the basis that they did not show significant differences (see ‘Statistical analysis’ section) between non-carriers and symptomatic mutation carriers after correction for age, sex, education and total intracranial volume. Each event represents the transition of a biomarker from a normal level (as seen in non-carriers) to an abnormal level (as seen in symptomatic patients). The probability a biomarker measurement is normal is modelled as a Gaussian distribution, and estimated using data from non-carriers. The distribution of abnormal measurements is also modelled as a Gaussian distribution, but estimated by fitting a mixture of two Gaussians (Fonteijn *et al.*, 2012) to data from all mutation carriers: the first Gaussian models the distribution of normal measurements, and is kept fixed to the values estimated from non-carriers; the second Gaussian models the distribution of abnormal measurements, and is optimized using data from mutation carriers. The sequence of events was estimated in various population subgroups: all 211 mutation carriers; 163 *PSEN1* mutation carriers; 17 *PSEN2* mutation carriers; and 31 *APP* mutation carriers. We also considered separate event-based models specific to *APOE* ɛ4 status: 61 mutation carriers who were *APOE* ɛ4-positive (with one or more *APOE* ɛ4 alleles), and 150 mutation carriers who were *APOE* ɛ4-negative. For further details of the model fitting procedures, see the ‘Statistical analysis’ section. We assigned participants to patient stages based on their most probable position along the most probable event sequence (Young *et al.*, 2014) for all mutation carriers combined. We assessed the efficacy of the patient staging system using only participants with longitudinal data available for all biomarkers (*n = *30, total of 42 follow-up visits), as missing entries cause uncertainty in a participant’s model stage.

#### Longitudinal: differential equation models

Reconstruction of biomarker trajectories ideally requires dense longitudinal data collected over the full time course of the disease. Such data are not yet available due to the prohibitive expense and complexity of collection, which means that we must resort to alternative methods. In dominantly-inherited Alzheimer’s disease and other neurodegenerative diseases, the availability of short-term longitudinal data of a few years permits estimation of an individual’s rate of change over that time span, e.g. via linear regression. These short-interval longitudinal observations are interpreted as noisy samples (segments) from an average biomarker trajectory. Instead of attempting to align the raw data segments (Donohue *et al.*, 2014), the differential equation modelling approach (Villemagne *et al.*, 2013; Oxtoby *et al.*, 2014) generates a cross-section of differential data and a model fit: biomarker rate-of-change as a function of biomarker value, i.e. a differential equation. For sufficient coverage across a range of biomarker values tracking disease progression, the fitted function can be integrated to produce a trajectory. We fit each biomarker in turn using a non-parametric Bayesian approach, and we aligned participants to a disease stage (time to onset) based on their biomarker measurements and the estimated probabilistic trajectories (see ‘Statistical analysis’ section).

### Statistical analysis

For fitting the event-based model we followed the same procedures as in Young *et al.* (2014). Briefly, the characteristic sequence and its uncertainty are estimated through a Markov chain Monte Carlo sampling procedure with greedy-ascent initialization for maximizing the data likelihood (Fonteijn *et al.*, 2012). We used a non-informative uniform prior on the sequence. When fitting an event-based model, it is important to select a set of biomarkers specific to the disease. That is, where disease ‘signal’ is present: a quantifiable distinction between normal and abnormal. For this procedure we used a paired *t*-test, and thresholded significance at *P* < 0.01/24, Bonferroni-corrected for multiple comparisons. We accounted for missing data as in Young *et al.* (2015) by imputing biomarker values such that missing measurements had an equal probability of being normal or abnormal. This ensures that the imputed data do not influence the characteristic sequence, while simultaneously allowing the subsets of available (non-missing) data to aid in elucidating the ordering among those subsets of biomarkers (*c.f.*Fig. 1 and Supplementary Fig. 16, with and without imputation of missing data). The maximum amount of missing data per biomarker that were imputed this way was 30% (PiB-PET), and the minimum was 7% (MMSE) (Table 1). We performed cross-validation of the event-based model by re-estimating the event distributions and maximum likelihood sequence for 100 bootstrap samples. The positional variance diagrams for the cross-validation results show the proportion of bootstrap samples in which event *i* (vertical axis) appears at position *k* (horizontal axis) of the maximum likelihood sequence.

For fitting differential equation models, we use a non-parametric approach known as Gaussian process regression (Rasmussen and Williams, 2006) to produce a probabilistic fit (a distribution of curves) that is determined by the data. The fitting was implemented within the probabilistic programming language Stan (Carpenter *et al.*, 2017), which performs full Bayesian statistical inference using Markov chain Monte Carlo sampling and penalized maximum likelihood estimation. We used a vanilla squared-exponential kernel (Rasmussen and Williams, 2006) for the Gaussian process prior covariance:

(1) $$k_{i,j}\left(x\right)=\eta^{2}\exp\left[-\rho^{2}{\left(x_{i}-x_{j}\right)}^{2}\right]+\delta_{i,j}\sigma^{2}$$

with hyperparameters η, ρ, σ, and Kronecker delta function δ. The Gaussian process prior hyperparameters guide the shape of the regression function, and were also estimated from the data. Here we used weakly-informative broad half-Cauchy hyperparameter priors, and diffuse initial conditions to aid model identifiability. We performed 10-fold cross-validation (Supplementary material), and various posterior predictive checks to assess model quality and numerical convergence (Gelman *et al.*, 2014; Vehtari *et al.*, 2016). We used out-of-sample validation for the model-based prediction of symptom onset in participants whose data were not used to build the models.

In dominantly-inherited Alzheimer’s disease, biomarker trajectories are usually investigated as a function of estimated years to onset. This is an approximate proxy for disease progression time where zero is the estimated point of onset of clinical symptoms, based on familial age of onset such as that of an affected parent. Here we defined *t* = 0 at a data-driven canonical abnormal level: the median biomarker value for symptomatic participants in the DIAN cohort (first symptomatic visit only).

A quantity of clinical interest is the interval of time between normal and abnormal biomarker levels, which we refer to as the ‘abnormality transition time’, and define in a data-driven manner via median values for asymptomatic (canonically normal) and symptomatic (canonically abnormal as above) participants in the DIAN dataset. Our probabilistic approach produces an abnormality transition time distribution per biomarker. The cumulative probability of abnormality produces data-driven sigmoid-like curves, which we combine across biomarkers to estimate a temporal pattern of disease progression.

We estimated time to onset (disease stage) for each individual using a weighted average across biomarkers. Aligning each biomarker measurement to each biomarker trajectory produces a set of biomarker-specific times, each with a data-driven credible interval given by the horizontal spread of the probabilistic trajectory. We weighted by the inverse width of the credible interval, to assign lower influence to estimates with large uncertainty. Incomplete data were used, with missing values omitted from the weighted average.

## Results

First, we present our cross-sectional multimodal modelling of the fine-grained ordering of dominantly-inherited Alzheimer’s disease biomarker abnormality using an event-based model. We then present our longitudinal modelling of dominantly-inherited Alzheimer’s disease biomarker trajectories using differential equation models.

### Cross-sectional results: event-based models of biomarker abnormality sequences

Figure 1 is a positional variance diagram of the maximum likelihood sequence of biomarker abnormality events (top to bottom), and its uncertainty (left to right), across all available 211 mutation carriers in the DIAN dataset. Greyscale intensity represents confidence in each event’s position within the sequence, and is calculated from Markov chain Monte Carlo samples from the event-based model (Young *et al.*, 2014). The closer this diagram is to a black diagonal, the more confidence there is in the disease progression sequence.

The event-based model reveals a distinct sequence of biomarker abnormality in dominantly-inherited Alzheimer’s disease: regional (cortical then striatal) amyloid deposition on PiB-PET scans; CSF measures of neuronal injury (total tau), neurofibrillary tangles (phosphorylated tau), and amyloid plaques (amyloid-β_42_ and amyloid-β_40_/amyloid-β_42_ ratio); MRI measures of volume loss in the putamen and nucleus accumbens. Thereafter the ordering in which global cognition (MMSE score), FDG-PET hypometabolism, and other MRI measures become abnormal is less certain. We found relatively high uncertainty early in the ordering of these biomarkers (as reflected by the more diffuse grey blocks straying from the diagonal), with a return to lower uncertainty later in the ordering of regional volumes (more solid dark blocks along the diagonal). This pattern (Fig. 1, left) persists under cross-validation (Fig. 1, right). Supplementary Fig. 16 shows an event-based model estimated without imputation of missing data, which qualitatively supports that our missing data imputation method does not bias the estimated sequence of abnormality (see ‘Statistical analysis’ section).

**Figure 2:**
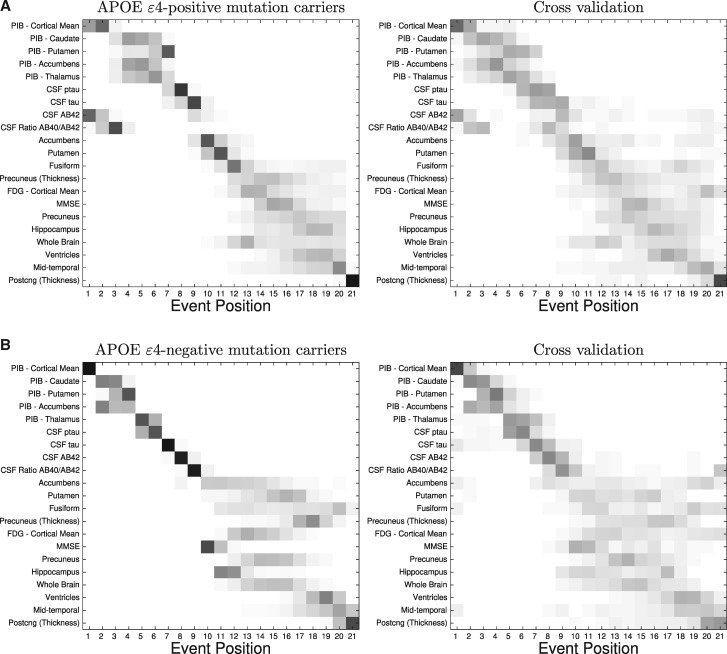
**Event-based models of dominantly-inherited Alzheimer’s disease: *APOE* ɛ4 groups.** Data-driven sequences of biomarker abnormality shown as positional variance diagrams for mutation carriers in the DIAN dataset who are: (**A**) *APOE* ɛ4-positive (*n* = 61); (**B**) *APOE* ɛ4-negative (*n* = 150). *C.f.*Fig. 1 (all groups combined): similar ordering, with a notable difference: *APOE* ɛ4-positive participants showed earlier CSF amyloid-β_42_ abnormality. AB = amyloid-β; Postcng = posterior cingulate; ptau = phosphorylated tau.

We also fit the event-based model to *APOE* ɛ4 subgroups of the mutation carriers in the dataset. Figure 2 shows positional variance diagrams of the biomarker abnormality event sequence in *APOE* ɛ4-positive and *APOE* ɛ4-negative participants (those with and without an apolipoprotein-4 allele). For ease of comparison, the sequence ordering on the vertical axes of each plot was chosen to be the most probable ordering from Fig. 1 (the result for all mutation carriers). Cross validation results are shown on the right of Fig. 2, as in Fig. 1.

Qualitatively, we see good agreement of the event sequences across *APOE* ɛ4 subgroups in Fig. 2, with notably earlier CSF amyloid-β_42_ and amyloid-β_40_/amyloid-β_42_ ratio in the *APOE* ɛ4-positive group.

We also performed an exploratory analysis of event-based models for mutation subtypes: Supplementary Fig. 1 shows positional variance diagrams of biomarker abnormality sequences in *PSEN1*, *PSEN2*, and *APP* mutation groups. While the numbers of participants in these groups may be too small to draw concrete conclusions about subtype differences (and the uncertainty is high in the orderings), we note some subtle differences: the *APP* subgroup shows earlier CSF amyloid-β abnormality; and the *PSEN2* subgroup shows earlier abnormality in the fusiform gyrus volume.

**Figure 3:**
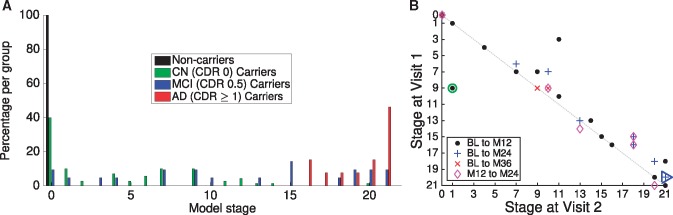
**Event-based model staging results for dominantly-inherited Alzheimer’s disease.** (**A**) Staging by diagnostic group: all non-carriers are at stage zero (black), and advancing disease stage is correlated strongly with cognitive impairment (green to blue to red). (**B**) Staging consistency across visits within 3 years of baseline for the *n* = 30 participants having complete longitudinal data (18 mutation carriers; 16 *PSEN1*, two *APP*). Most participants advance to a later stage (disease progresses towards the *right*). The green circle shows the single participant (a *PSEN1* mutation carrier) who regressed from event-based model stage 9 to stage 1, which arose due to discordant amyloid measurements between CSF and PiB-PET at baseline. The blue triangle indicates clinical progression from cognitively normal to MCI. AD = probable dementia due to dominantly-inherited Alzheimer's disease (global CDR > 0.5); BL = baseline; CN = cognitively normal (global CDR = 0); M = month; MCI = very mild dementia consistent with mild cognitive impairment (global CDR = 0.5).

Figure 3 demonstrates the fine-grained staging capabilities of the event-based model. Using the model for all mutation types (Fig. 1), each participant in the DIAN dataset was assigned a disease stage that best reflects their measurements (see ‘Materials and methods’ section, and Young *et al.*, 2014). The staging proportions are shown in Fig. 3A, differentiated by broad diagnostic groups defined using global CDR (CN: cognitively normal, global CDR = 0; MCI: very mild dementia consistent with mild cognitive impairment, global CDR = 0.5; AD: probable dementia due to Alzheimer’s disease, global CDR > 0.5). Longitudinal consistency of staging is shown in Fig. 3B where each participant’s baseline stage is plotted against available follow-up stages between baseline and months 12/24/36.

The baseline staging in Fig. 3A shows good separation of diagnostic groups: all of the non-carriers are assigned to stage 0 (black), presymptomatic mutation carriers (green) are predominantly at early model stages (with a notable exception—see ‘Discussion’ section), mutation carriers diagnosed with probable Alzheimer’s disease dementia are nearly all at late model stages, and mutation carriers with mild symptoms (CDR of 0.5) are more spread out across the stages. Within carriers, the model shows high classification accuracy for separating those who are cognitively normal from those with probable dementia: a balanced accuracy of 99% is achieved by classifying participants above stage 15 (MMSE abnormality) as having probable Alzheimer’s disease dementia. This shows that our generative model can also be used for discriminative applications with performance comparable to state-of-the-art multimodal binary classifiers (Willette *et al.*, 2014). Further, Supplementary Fig. 15A shows positive associations between familial estimates of years to onset and event-based model stage, by diagnostic group.

The follow-up staging in Fig. 3B shows good longitudinal consistency: at 33 of 36 (92%) follow-up time points the model stage is the same or it increased; at 35 of 36 (97%) follow-up time points the stage was either unchanged, it increased, or it decreased within the uncertainty of the ordering. This included the clinical converter shown with a blue triangle, whose CDR was 0 at baseline, and 0.5 at month 24. The follow-up time point at which the model stage decreased (green circle in Fig. 3B; a *PSEN1* mutation carrier) had inconsistent amyloid levels between CSF and regional PiB-PET, potentially due to discord between these biomarkers as has been observed in some individuals (Landau *et al.*, 2013; Schroeter *et al.*, 2015).

### Longitudinal results: biomarker trajectories from differential equation models

**Figure 4:**
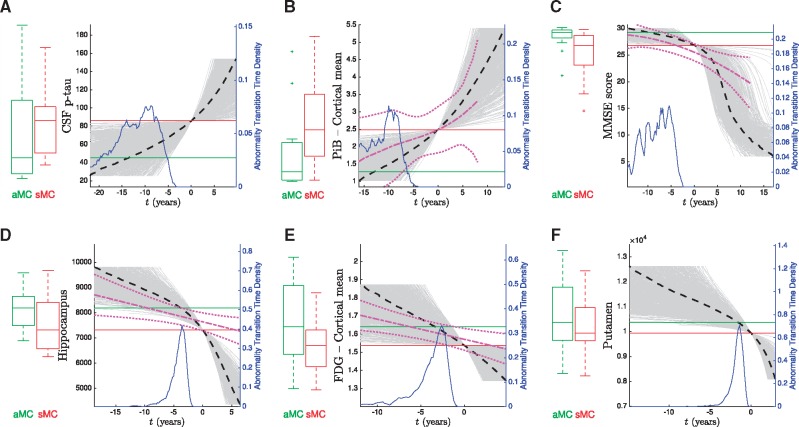
**Differential equation models: dominantly-inherited Alzheimer’s disease biomarker trajectories.** Shown are fits for selected biomarkers (see ‘Models’ section). Fits for other biomarkers are provided in the Supplementary material. Heavy black dashed lines show the average trajectory, with grey lines showing trajectories sampled from the posterior distribution. Time is expressed relative to the median biomarker value (red line) for symptomatic mutation carriers in the DIAN dataset (first visit with a non-zero CDR score), so that negative time suggests the average presymptomatic phase of dominantly-inherited Alzheimer’s disease. Box plots show biomarker distributions for asymptomatic (green, left, canonical normal) and symptomatic (red, right, canonical abnormal) mutation carriers (denoted aMC and sMC, respectively), with the distribution for estimated time between canonical normal and canonical abnormal (abnormality transition time) shown in blue. Details of included participants are given in Table 1. For comparison, the magenta fits in **B**–**E** are those from the linear mixed models of baseline DIAN data against estimated year of onset (EYO) from Bateman *et al.* (2012). SUVR = standardized uptake value ratio (relative to the cerebellum); p-tau = phosphorylated tau.

Figure 4 shows a selection of dominantly-inherited Alzheimer’s disease biomarker trajectories estimated from the DIAN dataset using our approach. Each average trajectory is shown as a heavy dashed black line, with uncertainty indicated by thin grey trajectories sampled from the posterior distribution. The time axis is defined such that t = 0 corresponds to the median biomarker value for symptomatic mutation carriers in the dataset, which we define as the canonical abnormal level. This is marked in Fig. 4 by a red horizontal line for each biomarker, with the corresponding distribution of biomarker values for symptomatic mutation carriers shown to the left of each trajectory as a red quartile box plot. The green quartile box plots show the biomarker distributions for asymptomatic mutation carriers, with the median value for each biomarker defining our canonical normal level and shown by a green horizontal line. Importantly, the canonical normal and abnormal levels are not required to estimate the biomarker trajectories, but are used to define the abnormality transition time for each biomarker as a data-driven estimate of the duration of the transition between these levels. Our Bayesian approach estimates an abnormality transition time density (probability distribution) for each biomarker, which is shown in blue in Fig. 4 (vertical axis on the right of each plot). For comparison, linear mixed model fits to baseline data from the same cohort in (Bateman *et al.*, 2012) are shown Fig. 4B–E (others not available).

Most trajectories in Fig. 4 (and in the Supplementary material) show acceleration from normal to abnormal levels, with little evidence for post-onset deceleration/plateauing that would be consistent with the sigmoidal behaviour hypothesized for sporadic Alzheimer’s disease in e.g. Jack *et al.* (2010). Biomarkers with trajectories that do not plateau, but remain dynamic into the symptomatic phase of the disease, offer potential utility for monitoring progression later in the disease. The grey curves capture uncertainty in the biomarker dynamics, which arises both from fitting the differential equation models to discrete data, and from heterogeneity in the population. For comparison with our data-driven approach, the magenta trajectories in Fig. 4B–E are from Bateman *et al.* (2012), which used regression of baseline data against estimated years to onset based on familial age of onset. Qualitatively, they broadly agree with our trajectories for PiB-PET (cortical average amyloid deposition), MMSE, hippocampus volume, and FDG-PET (cortical average hypometabolism), although, around symptom onset and beyond, our steeper hippocampus volume trajectory implies a more aggressive progression than estimated cross-sectionally in Bateman *et al.* (2012).

**Figure 5:**
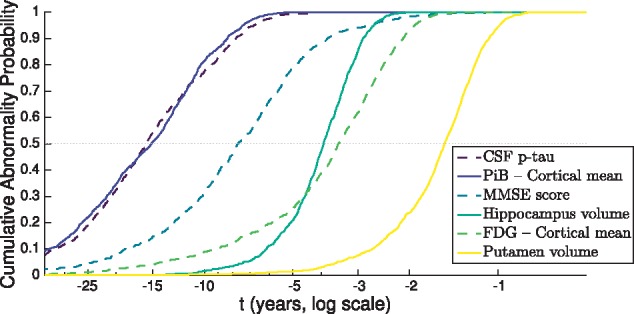
**Differential equation models: selected data-driven sigmoids for dominantly-inherited Alzheimer’s disease biomarker progression.** Cumulative probability of abnormality (vertical axis) is the empirical distribution of the abnormality transition time in years prior to canonical abnormality (horizontal axis) as per Fig. 4, calculated from each biomarker trajectory in Fig. 4. The horizontal axis shows years prior to canonical abnormality. The order of biomarkers in the legend follows the order in which they reach a cumulative probability of abnormality of 0.5 (horizontal dotted grey line). Green–blue–yellow colour scale (viridis) with alternating solid/dashed lines in order of cumulative abnormality probability reaching 0.5 (legend). p-tau = phosphorylated tau.

Figure 5 shows the cumulative probability for each biomarker in Fig. 4. That is, the empirical distribution function for the abnormality transition time densities in Fig. 4, using the same time axis but on a logarithmic scale to ease visualization. From Fig. 5 we can infer an ordering of abnormality by comparing the times at which each curve reaches an abnormality probability of 0.5.

The cumulative probability curves in Fig. 5 give a sense of both the average temporal ordering of biomarker abnormality (relative location of the curves at probability = 0.5), and the rate of progression (curve steepness) in the presymptomatic phase of dominantly-inherited Alzheimer’s disease. Whereas the event-based model approach is explicitly designed to infer an ordered sequence, our differential equation model approach is not. Nonetheless, the curves bear some resemblance to the hypothetical model in Jack *et al.* (2010), with the earliest phase of preclinical disease showing dynamic molecular pathology (CSF p-tau, and PiB-PET), and other biomarkers becoming dynamic as onset approaches: global cognitive decline (MMSE), neurodegeneration (MRI volumes), and hypometabolism (FDG-PET).

### Predicting time to symptom onset for unseen data

The models such as in Fig. 4 further support an estimated time from onset (together with uncertainty) for each biomarker—by aligning baseline biomarker measurement to the average trajectory. Uncertainty in each data-driven estimate of onset is given by the corresponding probabilistic trajectory distribution (grey curves in Fig. 4). A single estimate for each participant’s personal estimated time from onset, combining information from all biomarkers, then comes from averaging the estimates from each biomarker, weighted by inverse uncertainty. For validation, we compare our estimated time from onset to known actual years from onset for the six mutation carriers in the DIAN dataset who developed symptoms during the study (global CDR score becoming non-zero after baseline). These participants were omitted from the original differential equation model fits to avoid circularity.

**Figure 6:**
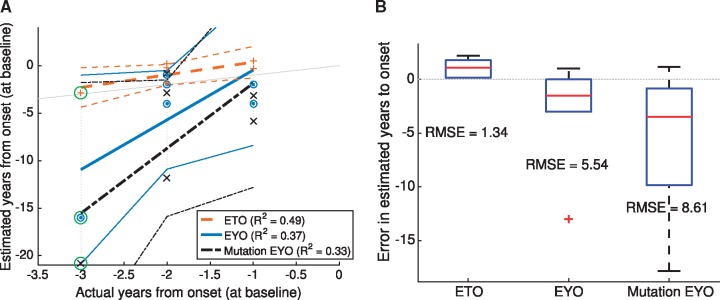
**Predicting onset of clinical symptoms.** For the six DIAN participants for whom global CDR became non-zero during the study (as of Data Freeze 6): (**A**) Estimated versus actual years to onset at baseline using our model-based approach and using familial age of onset (EYO) and mutation type age of onset (Mutation EYO). Mutation EYO is calculated from the average age of onset within the three mutation types, using data from Table e-1 in Ryman *et al.* (2014), with the average weighted by the number of affected individuals per mutation. The light grey line shows perfect correlation as a reference and participants’ data points are connected by dotted grey vertical lines. Our model-derived ETO (red asterisks and dashed line fit) correlates with actual years to onset better than familial EYO (blue triangles and solid line fits), as shown by the adjusted coefficient of determination (R^2^). The green circle highlights an individual for whom our approach (ETO) is superior to the traditional approach (EYO) for predicting years to onset. (**B**) Quartile boxplots of the error in predicting onset using each estimate: ETO (*left*) has a superior root-mean-squared error (RMSE) to both EYO (*middle*) and Mutation EYO (*right*), and predicts symptom onset to occur sooner rather than later, which is likely to be more accurate due to interval censoring (symptom onset occurring between visits to the clinic). ETO = estimated time from onset; EYO = estimated years from onset; RMSE = root mean squared error.

Figure 6A plots estimated years from onset against actual years from onset for our model-derived estimated time from onset (red asterisks; dashed line fit), and for familial estimates of years to onset (blue triangles; solid line fit), based on familial age of onset reported for an affected parent. A light grey line of reference shows perfect correspondence. Figure 6B shows quartile boxplots of the actual errors in predicting years to onset using our model-based approach and using familial age of onset, at the visit where progression occurred.

The linear fits in Fig. 6A and boxplots in Fig. 6B show that our data-driven estimated time from onset is a good predictor of actual years to onset with a root mean squared error of 1.34 years and a coefficient of determination of R^2^ ≈ 0.49. Familial age of onset is not as good: root mean squared error of 5.54 years and R^2^ ≈ 0.37 (based on parental age of onset); root mean squared error of 8.61 years and R^2^ ≈ 0.33 (based on mutation type). This poor performance is primarily because of very poor prediction for the participant at 3 years from onset (green circles, *PSEN1* mutation carrier). It is apparent from Fig. 6 that our estimated time from onset may tend to overestimate when onset will occur (predicting earlier onset), and familial age of onset tends to underestimate it (predicting later onset). This warrants further investigation with more data, but since onset may occur between visits to the clinic (interval censoring), it is likely more accurate to predict earlier onset, as our approach does.

### Overview of results

**Figure 7:**
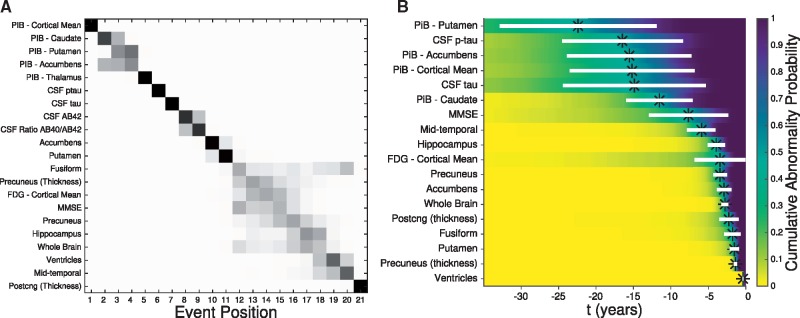
**Summary: data-driven models of dominantly-inherited Alzheimer’s disease progression.** (**A**) Event-based model for all mutation carriers in the DIAN, from Fig. 1. Biomarkers (imaging, molecular, cognitive) along the vertical axis are ordered by the maximum likelihood disease progression sequence (from *top* to *bottom*). The horizontal axis shows variance in the posterior sequence sampled using Markov chain Monte Carlo, with positional likelihood given by greyscale intensity. (**B**) Differential equation models. Each model-estimated biomarker trajectory (Fig. 4 and Supplementary Figs 5–7) estimates a probabilistic Abnormality Transition Time (years from canonical normal to canonical abnormal) and corresponding cumulative/empirical probability of abnormality (Fig. 5). Biomarkers along the vertical axis are ordered by the estimated sequence in which they reach 50% cumulative probability of abnormality (black asterisks). The viridis colour scale shows cumulative probability of abnormality increasing from the left (normal, yellow) to the right (abnormal, blue) as a function of years prior to canonical abnormality. White horizontal bars show the interquartile range of the abnormality transition time density, which visualizes the rate and duration of biomarker progression. p-tau* = *phosphorylated tau.

Figure 7 visualizes consistency across our two data-driven biomarker modelling approaches by showing patterns of dominantly-inherited Alzheimer’s disease progression obtained from each method on the DIAN dataset. The event-based model infers a probabilistic ordering of biomarker abnormality events through comparison of a cross-section of multi-modal observations, as shown for all mutation carriers in Fig. 7A (reproduced from Fig. 1). In contrast, each differential equation model works on an individual biomarker to estimate the biomarker trajectory. Figure 7B shows an alternative visualization of data-driven sigmoids for all included biomarkers, with the ordering determined as in Fig. 5 by cumulative abnormality probability reaching 0.5 (black asterisks; white bars indicate the speed of biomarker change—see Fig. 7 for details). Qualitatively, Fig. 7 shows that the different approaches estimate similar patterns of dominantly-inherited Alzheimer’s disease progression: accumulation of molecular pathology (amyloid, and tau where measured) followed by a blurring of cognitive abnormalities, brain hypometabolism, and regional changes to brain volume and cortical thickness. The combination of both models enables both a principled estimate of the sequence of biomarker abnormality, and temporal estimates of years to symptom onset.

## Discussion

In this section we discuss our results further and highlight new findings that warrant further investigation. To summarize, we report data-driven estimates of dominantly-inherited Alzheimer’s disease progression using two modelling approaches without reliance upon familial age of onset as a proxy for disease progression. The models reveal probabilistic sequences of biomarker abnormality from cross-sectional data across mutation groups, and probabilistic estimates of biomarker trajectories from a cross-section of short-term longitudinal data. The sequences and timescales broadly agree with current understanding of dominantly-inherited Alzheimer’s disease, while producing superior detail and predictive utility than previous work.

We take this opportunity to point out to the reader a caveat for any biomarker-based *in vivo* investigation of disease, model-based or otherwise: it is inherently limited by the precision and specificity of each biomarker. For example, our use of MMSE score as a cognitive biomarker limits us to making inferences about global cognitive decline, and not specific cognitive domains.

### Cross-sectional: event-based models

The event-based model finds a distinct ordering of biomarker abnormality events in mutation carriers (Fig. 1): amyloid deposition measured by PiB-PET, neurofibrillary tangles and amyloid plaques in CSF, followed by a pattern of regional volume loss on MRI that is characteristic of Alzheimer’s disease, which is interspersed with declining cognitive test scores and hypometabolism measured by FDG-PET. Although the sequence shows qualitative agreement across different mutation types (*PSEN1*, *PSEN2*, *APP*: Supplementary Fig. 1), and *APOE* ɛ4 carrier groups (positive and negative: Fig. 2), we found some small, subtle differences that warrant further investigation. For example, there was earlier abnormality in CSF amyloid-β_42_ (than CSF tau) in the *APP* and *APOE* ɛ4-positive groups, but the reverse was found in other groups. The latter could be explained by non-monotonic dynamics of CSF amyloid-β_42_ markers in dominantly-inherited Alzheimer’s disease (an increase followed by a decrease) as suggested by results in previous investigations (Reiman *et al.*, 2012; Fagan *et al.*, 2014), and consistent with our own differential equation modelling investigation (see below and Supplementary material). Previous multimodal biomarker studies of dominantly-inherited Alzheimer’s disease (Bateman *et al.*, 2012; Benzinger *et al.*, 2013; Fleisher *et al.*, 2015) are in general agreement with the event-based model sequence: amyloidosis precedes hypometabolism, neurodegeneration, and cognitive decline. Note that we considered cross-sectional volumes of brain regions, not direct measures of atrophy, which can explain why cognitive decline appears earlier than might be expected (Young *et al.*, 2014). Importantly, all previous approaches relied upon a familial age of symptom onset as a proxy for disease progression, which intrinsically limits the accuracy of predictions due to the known imprecision in such estimates (Ryman *et al.*, 2014). Further, such models cannot be easily generalized to sporadic forms of disease where no such proxy for disease progression exists, whereas ours can, e.g. event-based models of sporadic Alzheimer’s disease in Young *et al.* (2014). Having said that, we do not advocate quantitative application of models of familial disease directly on sporadic disease cases due to differences such as those seen in amyloid imaging between sporadic and familial Alzheimer’s disease (Bateman *et al.*, 2011).

The similarity of the event-based model sequence for dominantly-inherited Alzheimer’s disease with that for sporadic Alzheimer’s disease in previous work (Young *et al.*, 2014) supports the notion that these two forms of Alzheimer’s disease have similar underlying disease mechanisms, and therefore that drugs developed on dominantly-inherited Alzheimer’s disease may be efficacious in sporadic Alzheimer’s disease. We note some slight deviations of the dominantly-inherited Alzheimer’s disease sequence here from the sporadic Alzheimer’s disease sequence in Young *et al.* (2014): the involvement of the putamen, nucleus accumbens, precuneus and posterior cingulate. Other dominantly-inherited Alzheimer’s disease investigations have observed involvement of the precuneus and cingulate regions (Scahill *et al.*, 2002; Benzinger *et al.*, 2013; Cash *et al.*, 2013). Our earlier study of sporadic Alzheimer’s disease did not include these regions in the analysis, so further work will be required to determine their involvement in sporadic Alzheimer’s disease event-based models. Moreover, the nature of the biomarkers we use here means that we cannot determine whether sporadic Alzheimer’s disease and dominantly-inherited Alzheimer’s disease are similar on the microscopic scale.

The staging system provided by the event-based model has potential practical utility. In particular, it provides high classification accuracy for discriminating between presymptomatic and genuinely symptomatic (global CDR ≥ 1) dominantly-inherited Alzheimer’s disease mutation carriers. Although further work is necessary to determine whether model-based discrimination of subtle cognitive decline (CDR of 0.5 versus 0) is sufficiently accurate to have practical utility. Our staging system correctly assigned all non-carriers to the ‘completely normal’ category (stage 0), and shows good longitudinal consistency, with event-based model stage generally increasing or remaining stable at patient follow-up. This encourages us to suggest that the staging system has utility in future clinical trials, both for screening of potential participants and for defining end-points. For example, recruiting individuals at event-based model stages 1–5 (Fig. 1: PiB-PET abnormality only), and defining an end-point as reaching stage 8 (addition of CSF abnormality). The same approach could work for personalized treatment assignment. For example, an anti-amyloid agent might only be appropriate for *APOE* ɛ4-positive individuals at event-based model stages 1 and 2 (Fig. 2A).

We found the event-based model stages to correlate strongly with cognitive status (Fig. 3A): cognitively normal participants were assigned early model stages, symptomatic dominantly-inherited Alzheimer’s disease participants were assigned late model stages, and participants with mild symptoms were more spread out across the stages. The mildly symptomatic group in dominantly-inherited Alzheimer’s disease were the most heterogeneous, which is in agreement with our results in sporadic Alzheimer’s disease (Young *et al.*, 2014), but possibly for different reasons. One contributing factor in dominantly-inherited Alzheimer’s disease is that the mildly symptomatic group may include unaffected mutation carriers whose anxiety about their mutation status manifested as apparent cognitive abnormality and contributed to their diagnosis (global CDR of 0.5). Another possibility is that cognitive reserve may play a role, given the younger age of the cohort than is typical of sporadic Alzheimer’s disease. In any case, the fine-grained disease staging offered by the event-based model can shed light upon the heterogeneity contained within a prodromal disease stage. Separate work will consider explicitly modelling prodromal disease phases within the event-based model. The most notable outlier in our staging analysis was an asymptomatic individual (green bar at stage 20 in Fig. 3A), who was assigned an advanced model stage of 20 (maximum 21) at baseline. This individual had 17 of 21 biomarkers with abnormal measurements, but no apparent symptoms (global CDR of 0) until 24 months later when their global CDR was 0.5 and model stage was 21 (blue triangle in Fig. 3B). Supplementary Fig. 15A shows that event-based model stage correlates with familial age of onset, although further follow-up will be required to ascertain the predictive utility of event-based model stage compared to familial age of onset by looking at a large number of individuals who develop clinical Alzheimer’s disease dementia during a study of dominantly-inherited Alzheimer’s disease.

### Longitudinal: differential equation models

Our non-parametric fits to differential biomarker data are data-driven probabilistic estimates of an underlying differential equation driving the disease biomarker evolution. Since there is no ground truth disease stage (e.g. time to symptom onset), the differential equation approach assumes a one-to-one mapping of biomarker value to disease progression in order to infer disease stage, which limits the approach to estimating only monotonic biomarker trajectories. Further, the use of a single differential data point per participant precludes modelling within-individual dynamics using this approach. The consequence is that if enough individuals display contrary dynamics to the average, perhaps due to measurement noise for example, then a sensible trajectory cannot be inferred. This happened for CSF markers of tau, amyloid-β_42_, and the amyloid-β_40_/amyloid-β_42_ ratio, as shown in Supplementary Fig. 14. Otherwise, we obtained trajectory estimates for the same set of biomarkers in the event-based model results (Fig. 4 and Supplementary material). Most differential equation model-estimated biomarker trajectories showed accelerating dynamics, with little or no apparent deceleration, which may arise from under-sampling of later disease stages (for example because recruitment in this cohort was focused on presymptomatic dominantly-inherited Alzheimer’s disease). The magenta fits in Fig. 4 correspond to those in Bateman *et al.* (2012) (taken directly from the Supplementary material in that paper), which was a cross-sectional regression of biomarker trends as a function of familial estimates of years to onset, in the DIAN dataset. It is apparent from Fig. 4 that the most noteworthy difference between the differential equation model trajectories and familial age of onset regression trajectories are the slower post-onset dynamics estimated for hippocampal volume when using the latter. The cross-sectional approach, such as in Bateman *et al.* (2012) and Benzinger *et al.* (2013), is less able to capture speed of progression than the differential equation modelling approach, which utilizes short-duration longitudinal data, within subjects. This is supported by the longitudinal analysis in the familial age-of-onset-based regional imaging biomarker investigation in Benzinger *et al.* (2013), which found that the cross-sectional biomarker trajectory tended to underestimate the slope of individual trajectories, post-onset.

We did not model biomarker measurement noise. Such noise can lead to regression dilution, which, in a differential equation modelling approach, would produce an elongated (slower) biomarker trajectory. Thus, temporal quantities we have estimated, such as abnormality transition times, may represent overestimates—particularly for biomarkers with large measurement noise. However, no regression dilution was apparent, as evidenced by our ability to accurately predict actual symptom onset (discussed below).

Our longitudinal analysis includes a step whereby non-progressing, normal biomarker measurements are excluded, as done in Villemagne *et al.* (2013). It could be argued that this approach might potentially lead to an overemphasis on change by removing some data points that are on the trajectory, but that appear stable (due perhaps to measurement noise). We feel that our results did not show this, as supported by our ability to predict symptom onset in unseen data.

Recently, Ryman *et al.* (2014) performed a meta-analysis of actual symptom onset in multiple studies of dominantly-inherited Alzheimer’s disease including the DIAN, and considered prediction of age at symptom onset using ages of onset for parents, family average, and group-wise averages by mutation type, as well as *APOE* ɛ4 genotype and sex. They argued that mutation type and family history should be used to estimate onset in clinical research. This conclusion was reached by analysing the proportion of variance in actual age of onset that could be explained by these factors in a linear regression scenario, quantified by adjusted R^2^. Specifically, they found R^2^ = 0.3838 (parental), R^2^ = 0.4906 (family average) and R^2^ = 0.5225 (mutation type). For clinical utility we argue that a model’s predictive accuracy should be quantified, such as by using root mean squared error in prediction of unseen data. We quantified predictive accuracy for six participants in the DIAN dataset with observed symptom onset (at Data Freeze 6) in Fig. 6—we found root mean-squared error of 5.54 years with R^2^ = 0.37 (parental), and root mean squared error of 8.61 years with R^2^ = 0.33 (mutation type), whereas our data-driven model-based approach performed considerably better: root mean squared error of 1.35 years with R^2^ = 0.44.

## Conclusion

Dominantly-inherited Alzheimer’s disease progression occurs over multiple decades. Our two data-driven approaches have estimated dominantly-inherited Alzheimer’s disease progression models by combining shorter cross-sections of data. This was made possible in part by assuming a single progression pattern across individuals. Despite this, our models are able to predict probabilistic outcomes for individuals by comparing them to the average pattern. With increased availability of data, especially actual symptom onset, an important future aim is to incorporate multilevel modelling to improve the specificity of predictions across mutation types, families, and individuals, and to hopefully understand more of the heterogeneity observed in dominantly-inherited Alzheimer’s disease.

Our probabilistic, data-driven computational models of dominantly-inherited Alzheimer’s disease reveal evidence-based patterns in the progression of this relatively rare disease. The similarities with sporadic Alzheimer’s disease progression provides encouragement for ongoing trials into anti-amyloid therapies such as the ones currently underway by the DIAN Trials Unit. We have also demonstrated abilities of the data-driven models for fine-grained patient staging and prognosis, which promises utility for recruitment, stratification, and surrogate outcome measures in clinical trials.

## Supplementary Material

Supplementary Data

## Abbreviations

CDR: Clinical Dementia Rating
DIAN: Dominantly Inherited Alzheimer Network
MMSE: Mini-Mental State Examination
PiB: Pittsburgh compound B
SUVR: standardized uptake value ratio
