# Supplementary material for: Data-driven models of dominantly-inherited Alzheimer’s disease progression
Source: Brain. 2018 Mar 22;141(5):1529–44. doi: 10.1093/brain/awy050 (PMC5920320; doi:10.1093/brain/awy050)
Supplement: Supplementary Data [file awy050_brain-2017-00936-file004.pdf]

Supplementary Material

Supplementary Figure 1 shows an exploratory analysis of event-based models of dominantly-inherited Alzheimer's disease mutation subtypes: Presenilin 1, Presenilin 2, and Amyloid Precursor Protein.

Differential equation model fits for selected biomarkers in the Dominantly Inherited Alzheimer Network dataset which displayed monotonic behaviour on average are shown in Supplementary Figure 2 (cross-validation in Supplementary Figure 8), Supplementary Figure 3 (cross-validation in Supplementary Figure 9), and Supplementary Figure 4 (cross-validation in Supplementary Figure 10). Corresponding biomarker trajectories are shown in Supplementary Figure 5 (cross-validation in Supplementary Figure 11), Supplementary Figure 6 (cross-validation in Supplementary Figure 12), and Supplementary Figure 7 (cross-validation in Supplementary Figure 13), respectively.

Supplementary Table 1 is a numerical summary of the differential equation model fitting results: the model hyperparameter estimates and numerical convergence of the Markov chain Monte Carlo fits via the potential scale reduction factor $\hat{R}$ (Gelman *et al.*, 2014; Vehtari *et al.*, 2016). Ten-fold cross-validation results are quoted as mean ± standard deviation across the ten folds.

Supplementary Figure 14 shows differential equation model fits for the few CSF amyloid biomarkers where the approach was incapable of estimating valid biomarker trajectories due apparently to non-monotonic dynamics.

Supplementary Figure 15 compares our model-based measures of dominantly-inherited Alzheimer's disease progression to the traditional measure, Estimated Years to Onset

(denoted EYO in the figure) based on parental age of onset. Further discussion of this is included below.

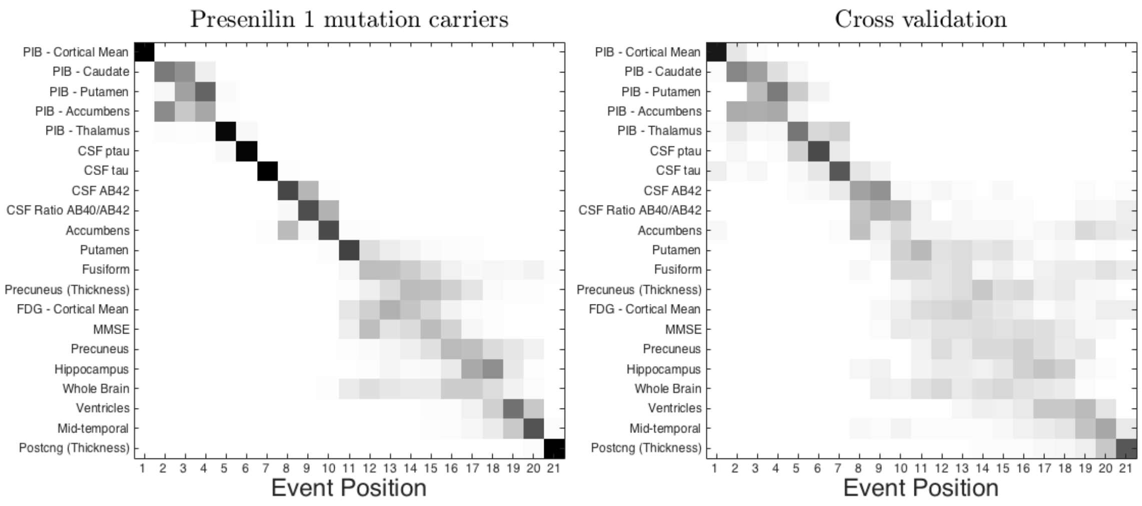

A. Presenilin 1 mutation carriers (n=163)

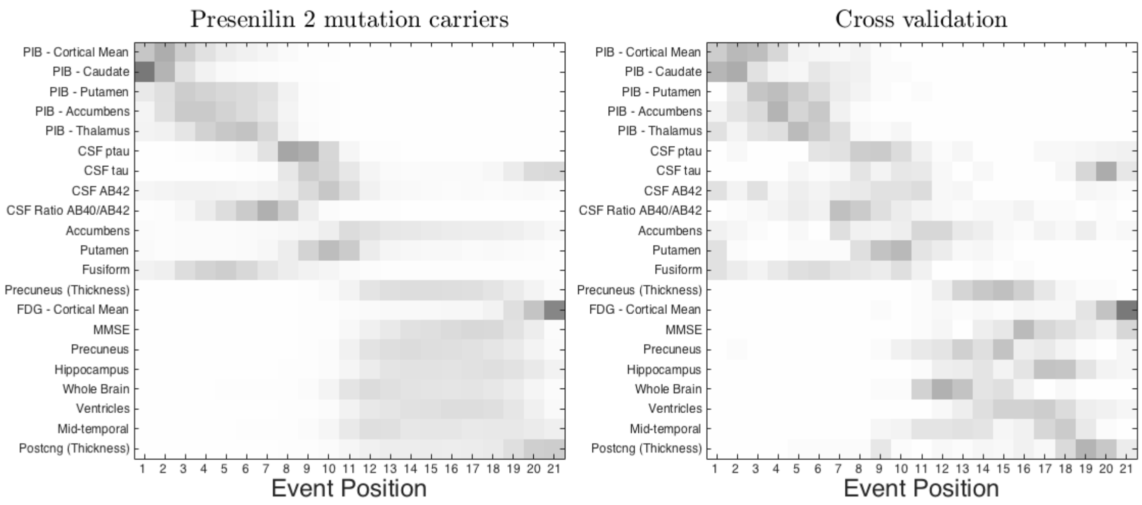

B. Presenilin 2 mutation carriers (n=17)

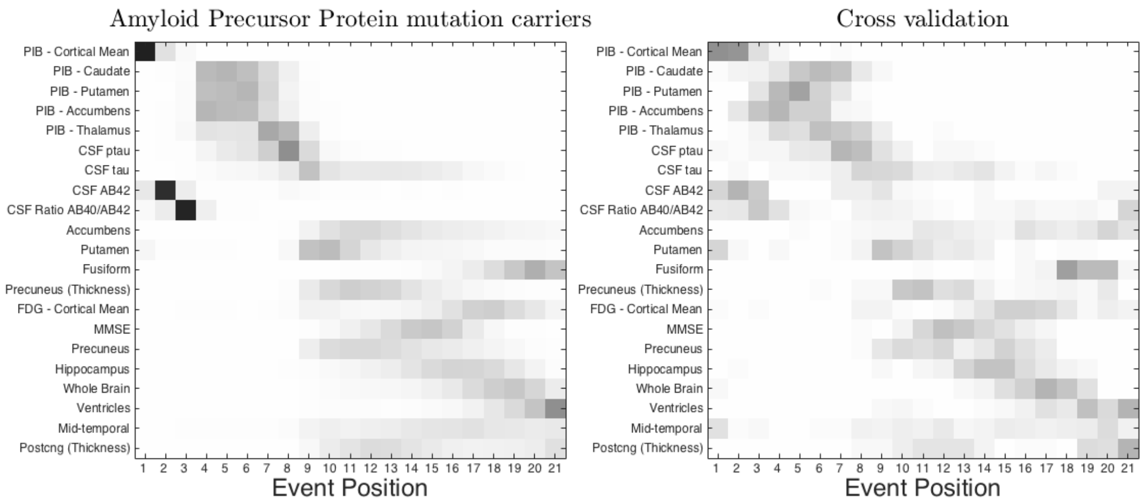

C. Amyloid Precursor Protein mutation carriers (n=31)

*Supplementary Figure 1. **Event-based models of dominantly-inherited Alzheimer's disease: mutation type subgroups.** Data-driven sequences of biomarker abnormality shown as positional variance diagrams for mutation carriers in the DIAN dataset who are A. Presenilin 1 mutation carriers; B. Presenilin 2 mutation carriers; C. Amyloid Precursor Protein mutation carriers. Compare with **Error! Reference source not found.** (all groups*

combined): similar ordering, with a notable difference: Amyloid Precursor Protein mutation carriers showed earlier CSF Aβ42 abnormality. Abbreviations as in ***Error! Reference source not found.****.*

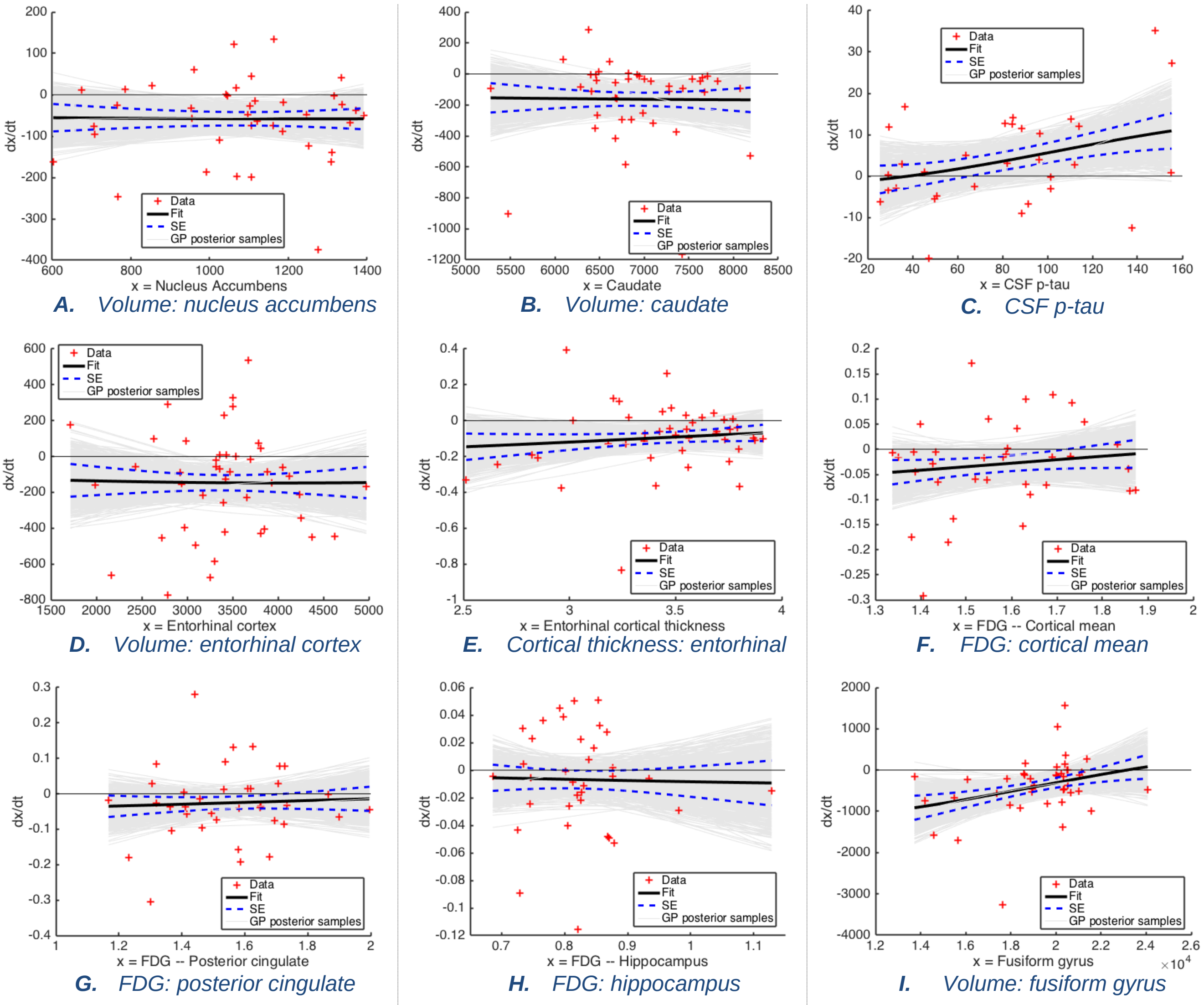

Supplementary Figure 2. Differential equation model fits (1 of 3). Data points are shown as red plusses. The Bayesian nonparametric differential regression fits are shown as mean (heavy solid black line) ± standard error (SE, dashed blue lines), and samples from the full posterior (light grey lines). Gaussian process model hyperparameters are in Supplementary Table 1. Corresponding biomarker trajectories are shown in Supplementary Figure 5.

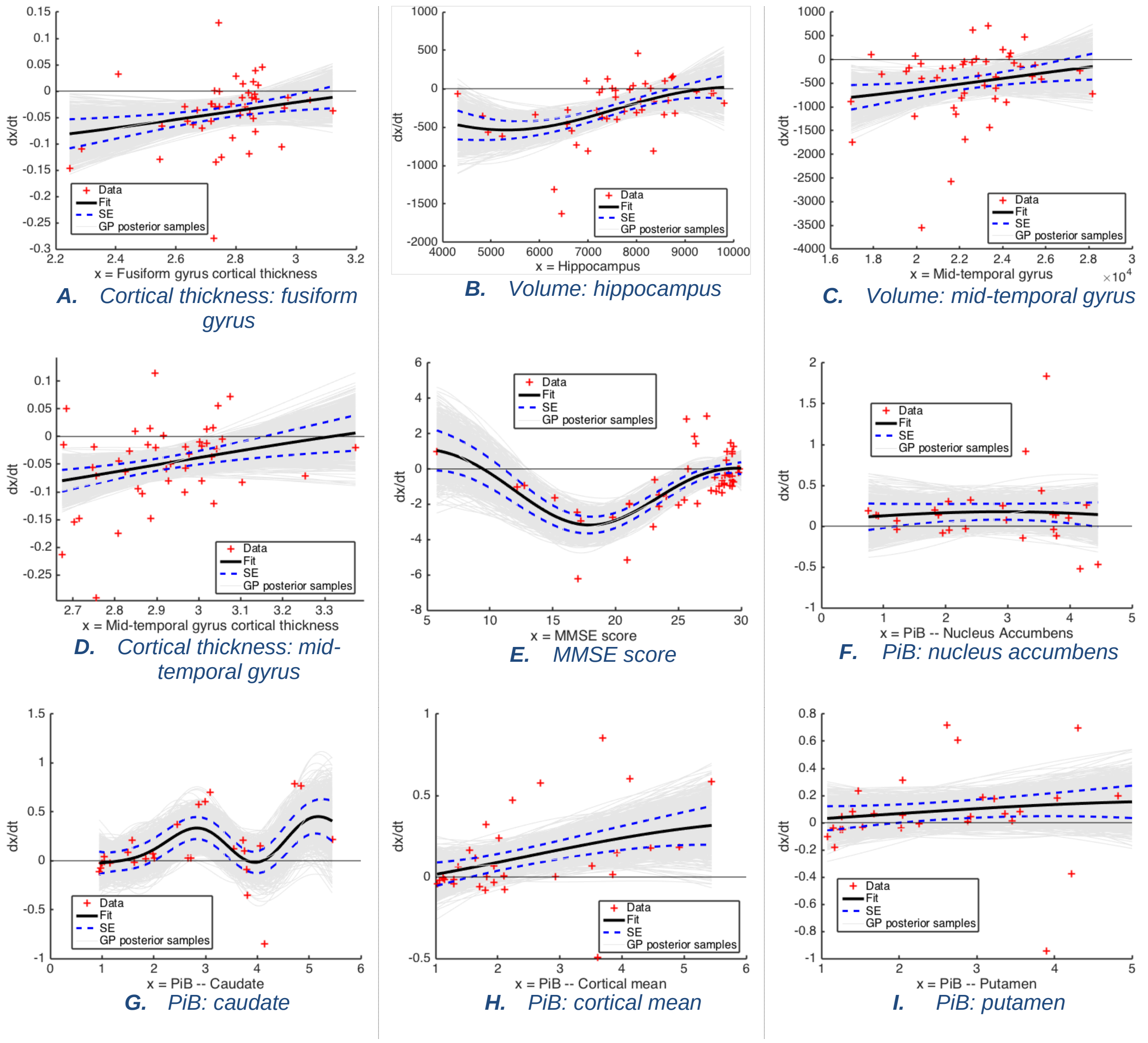

A. *Cortical thickness: fusiform gyrus*

B. *Volume: hippocampus*

C. *Volume: mid-temporal gyrus*

D. *Cortical thickness: mid-temporal gyrus*

E. *MMSE score*

F. *PiB: nucleus accumbens*

G. *PiB: caudate*

H. *PiB: cortical mean*

I. *PiB: putamen*

Supplementary Figure 3. Differential equation model fits (2 of 3). Corresponding biomarker trajectories shown in Supplementary Figure 6. Key explained in Supplementary Figure 2.

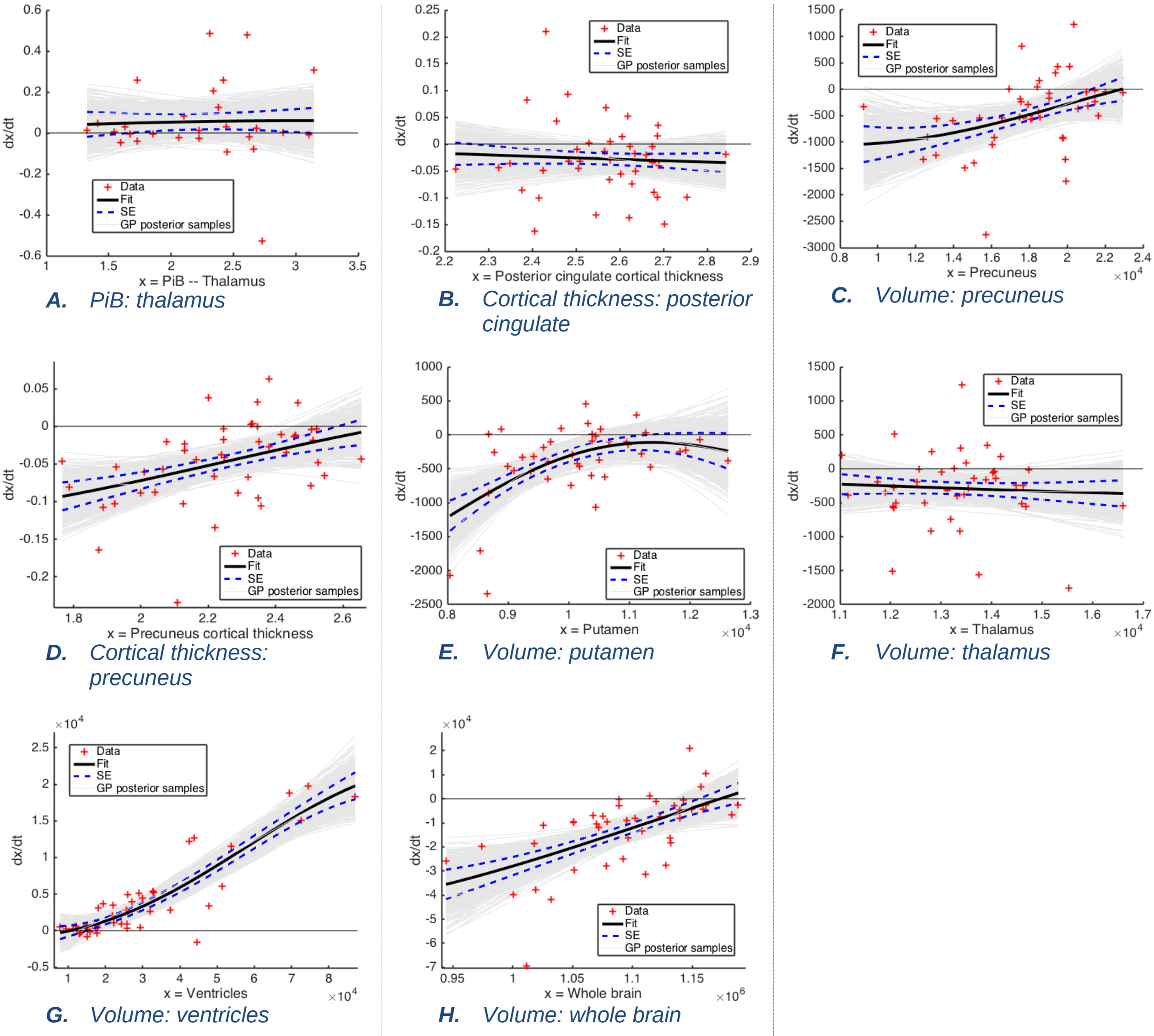

Supplementary Figure 4. Differential equation model fits (3 of 3). Corresponding biomarker trajectories shown in Supplementary Figure 7. Key explained in Supplementary Figure 2.

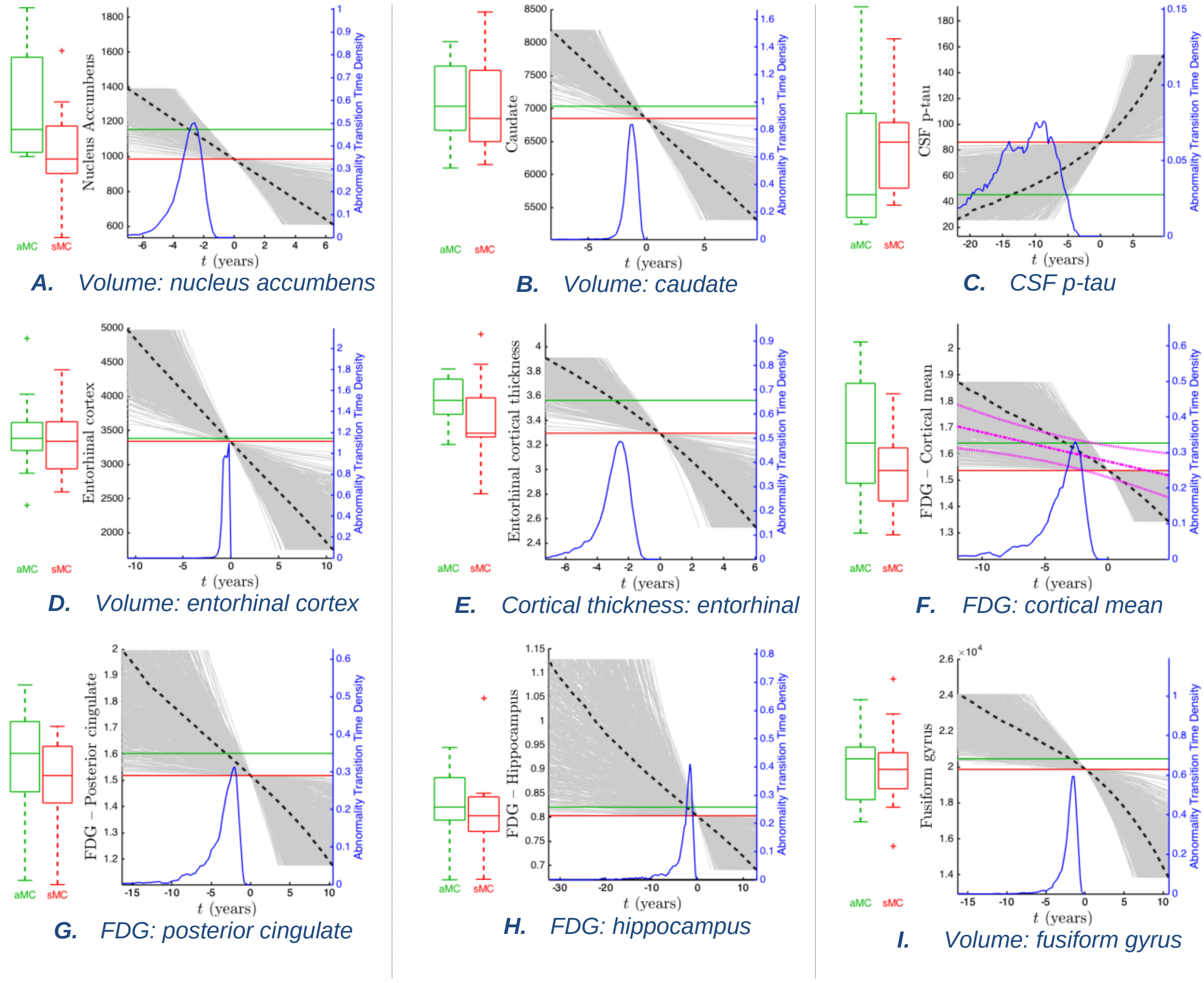

Supplementary Figure 5. Biomarker trajectories (1 of 3). Corresponding differential equation model fits shown in Supplementary Figure 2. Key explained in Error! Reference source not found..

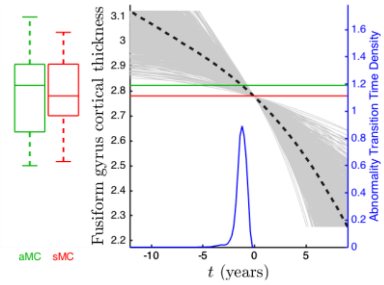

A. *Cortical thickness: fusiform gyrus*

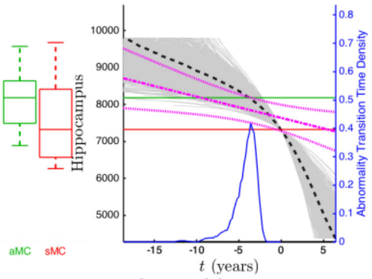

B. *Volume: hippocampus*

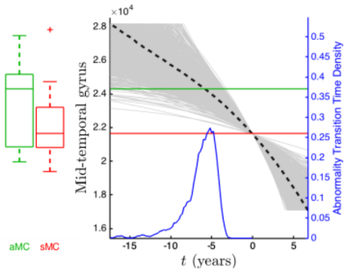

C. *Volume: mid-temporal gyrus*

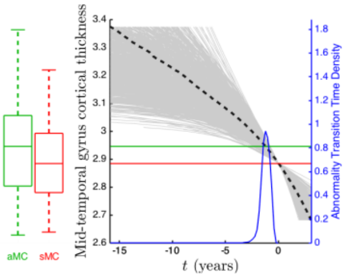

D. *Cortical thickness: mid-temporal gyrus*

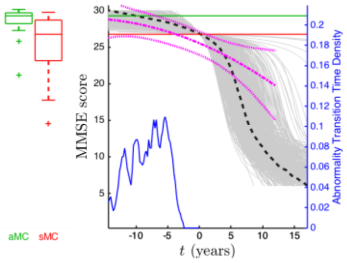

E. *MMSE score*

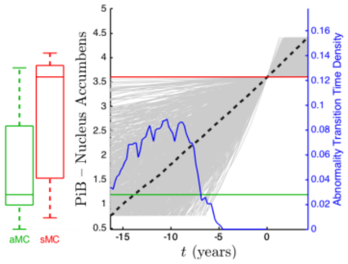

F. *PiB: nucleus accumbens*

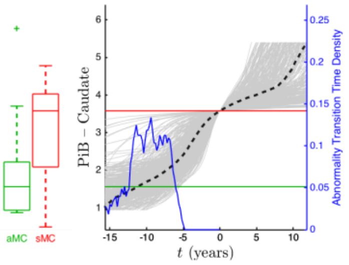

G. *PiB: caudate*

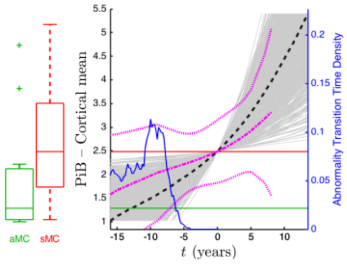

H. *PiB: cortical mean*

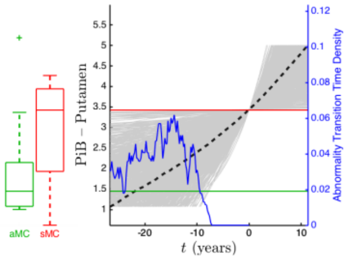

I. *PiB: putamen*

Supplementary Figure 6. Biomarker trajectories (2 of 3). Corresponding differential equation model fits shown in Supplementary Figure 3. Key explained in Error! Reference source not found..

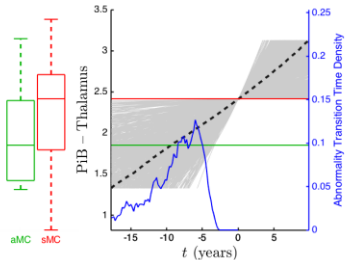

A. *PiB: thalamus*

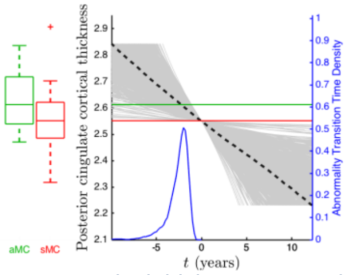

B. *Cortical thickness: posterior cingulate*

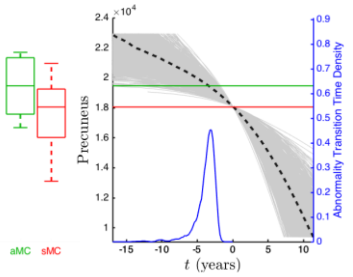

C. *Volume: precuneus*

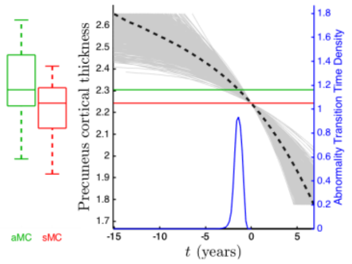

D. *Cortical thickness: posterior cingulate*

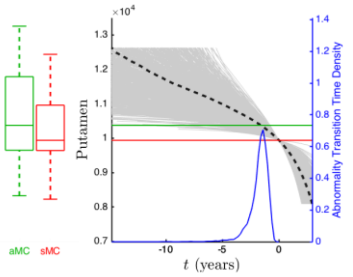

E. *Volume: putamen*

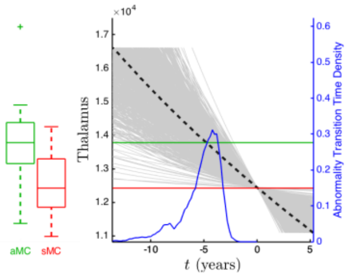

F. *Volume: thalamus*

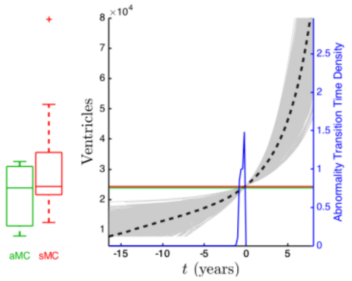

G. *Volume: ventricles*

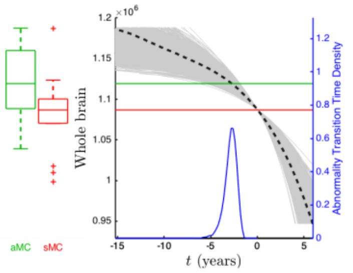

H. *Volume: whole brain*

Supplementary Figure 7. Biomarker trajectories (3 of 3). Corresponding differential equation model fits shown in Supplementary Figure 4. Key explained in Error! Reference source not found..

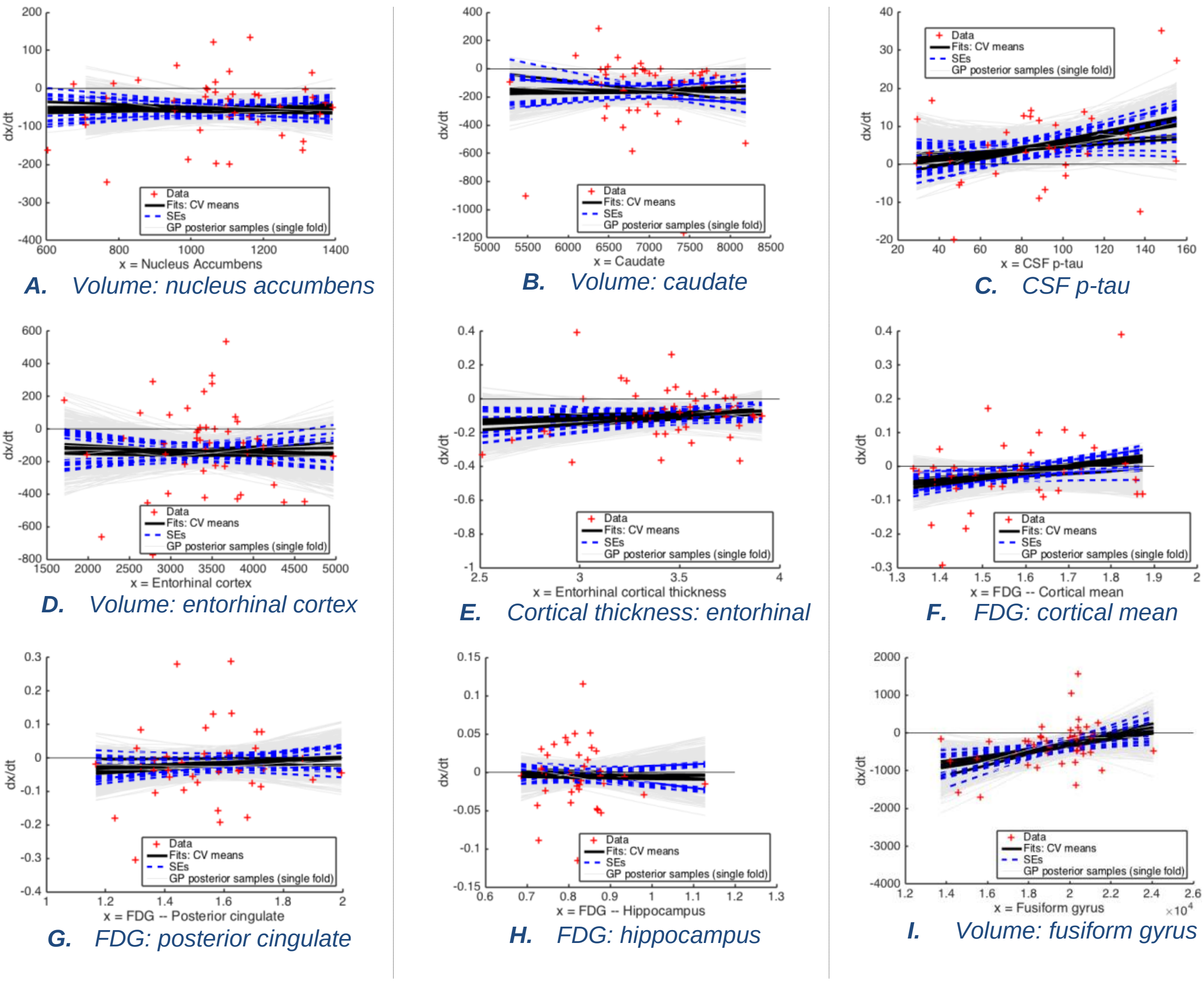

Supplementary Figure 8. Differential equation model fits: ten-fold cross-validation (1 of 3) of the fits in Supplementary Figure 2. Corresponding biomarker trajectories are in Supplementary Figure 11.

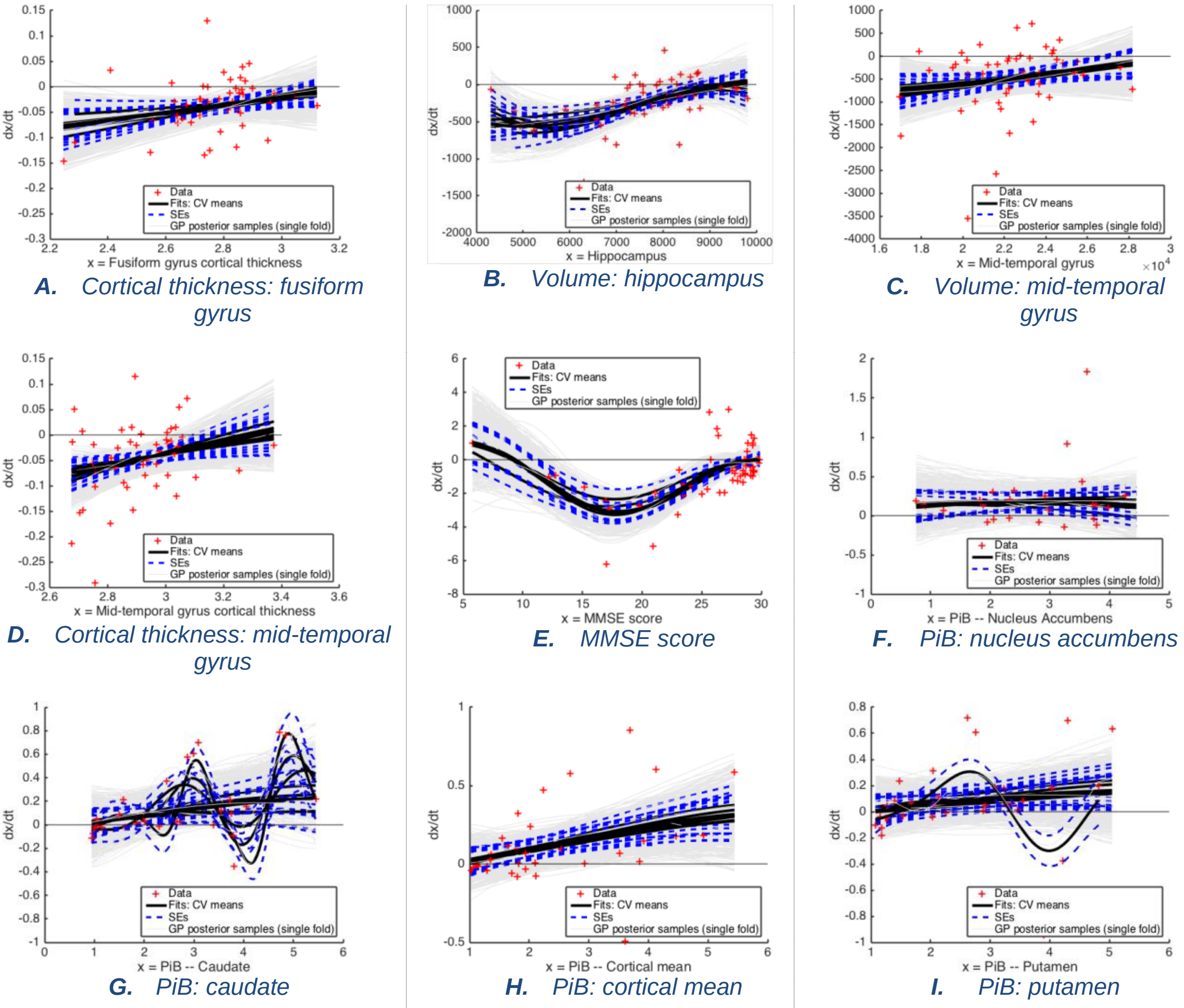

Supplementary Figure 9. Differential equation model fits: ten-fold cross-validation (2 of 3) of the fits in Supplementary Figure 3. Corresponding biomarker trajectories are in Supplementary Figure 12.

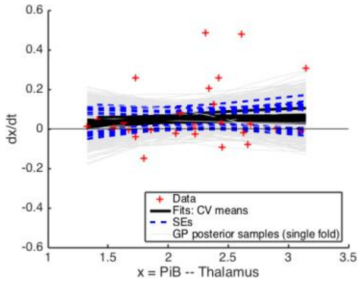

A. *PiB: thalamus*

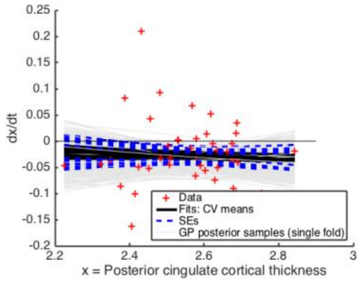

B. *Cortical thickness: posterior cingulate*

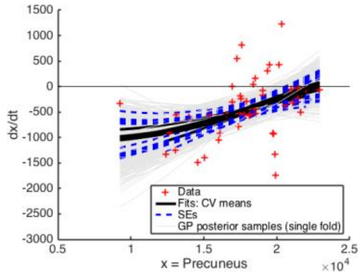

C. *Volume: precuneus*

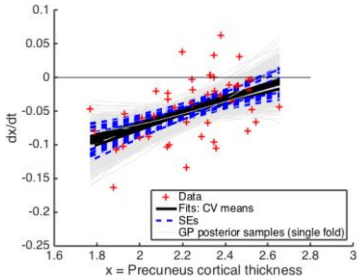

D. *Cortical thickness: posterior cingulate*

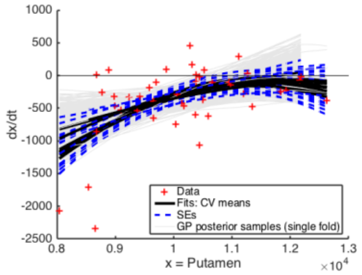

E. *Volume: putamen*

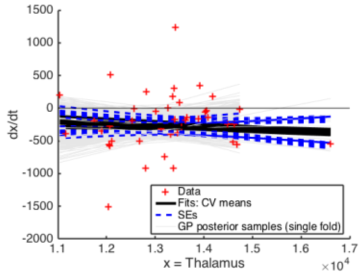

F. *Volume: thalamus*

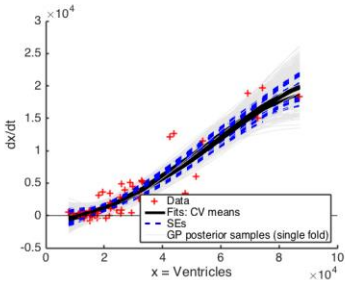

G. *Volume: ventricles*

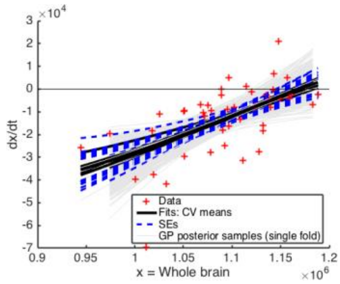

H. *Volume: whole brain*

Supplementary Figure 10. Differential equation model fits: ten-fold cross-validation: (3 of 3) of the fits in Supplementary Figure 4. Corresponding biomarker trajectories are in Supplementary Figure 13.

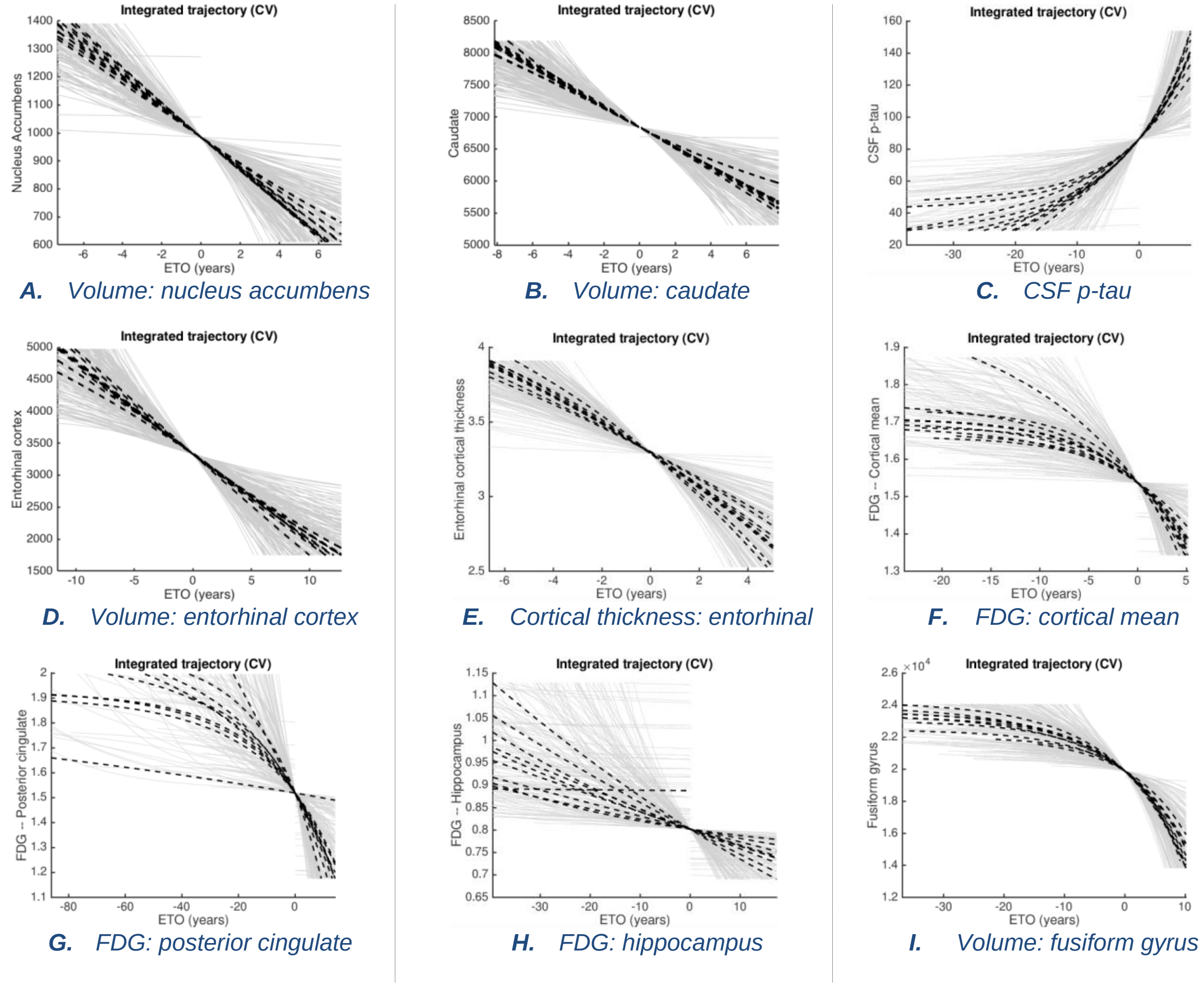

Supplementary Figure 11. Biomarker trajectories: ten-fold cross-validation (1 of 3). Corresponding differential equation model fits are in Supplementary Figure 8.

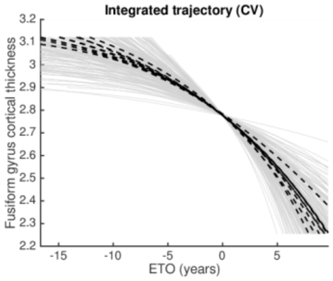

A. *Cortical thickness: fusiform gyrus*

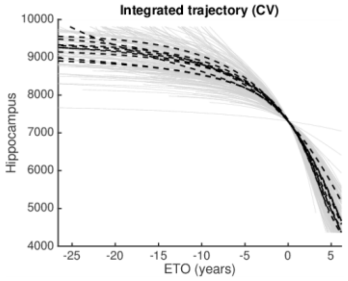

B. *Volume: hippocampus*

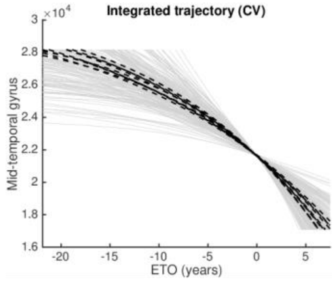

C. *Volume: mid-temporal gyrus*

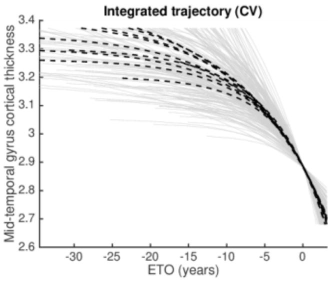

D. *Cortical thickness: mid-temporal gyrus*

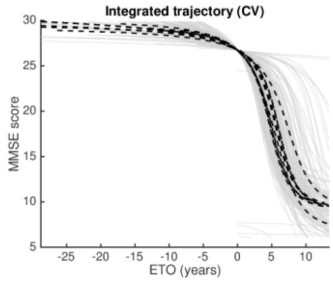

E. *MMSE score*

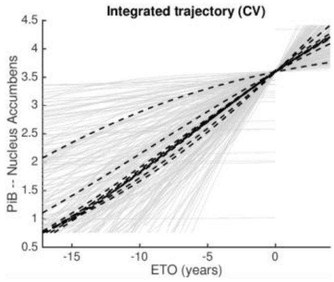

F. *PiB: nucleus accumbens*

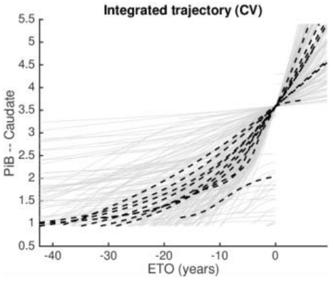

G. *PiB: caudate*

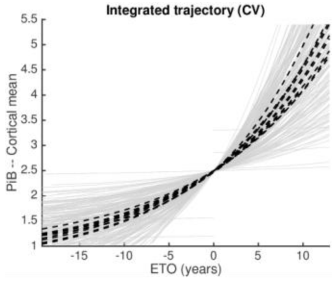

H. *PiB: cortical mean*

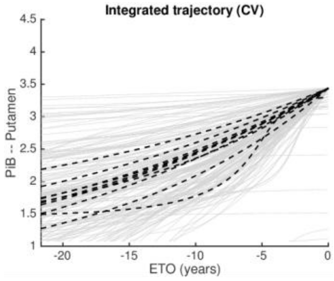

I. *PiB: putamen*

Supplementary Figure 12. Biomarker trajectories: ten-fold cross-validation (2 of 3). Corresponding differential equation model fits are in Supplementary Figure 9.

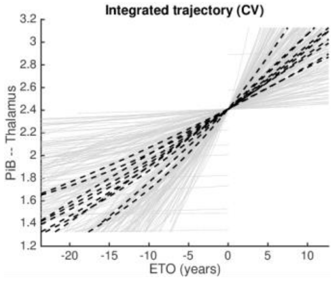

A. *PiB: thalamus*

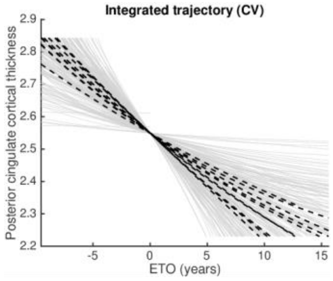

B. *Cortical thickness: posterior cingulate*

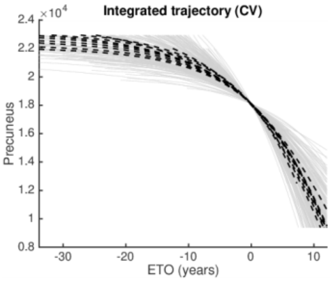

C. *Volume: precuneus*

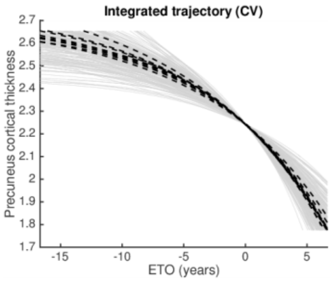

D. *Cortical thickness: precuneus*

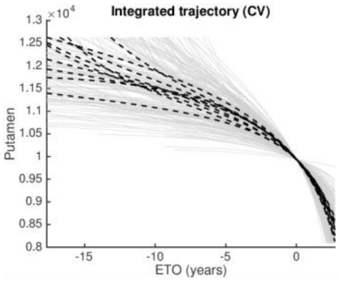

E. *Volume: putamen*

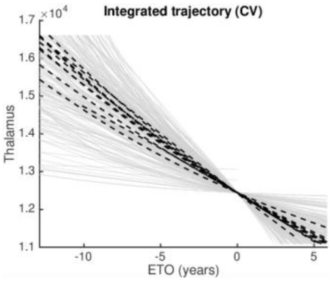

F. *Volume: thalamus*

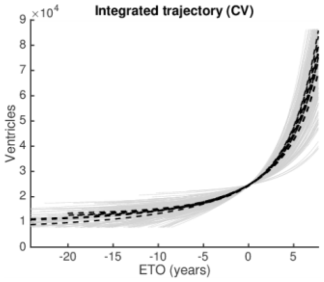

G. *Volume: ventricles*

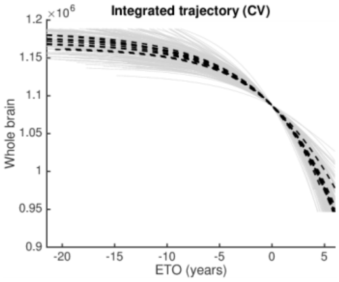

H. *Volume: whole brain*

Supplementary Figure 13. Biomarker trajectories: ten-fold cross-validation (3 of 3). Corresponding differential equation model fits are in Supplementary Figure 10.

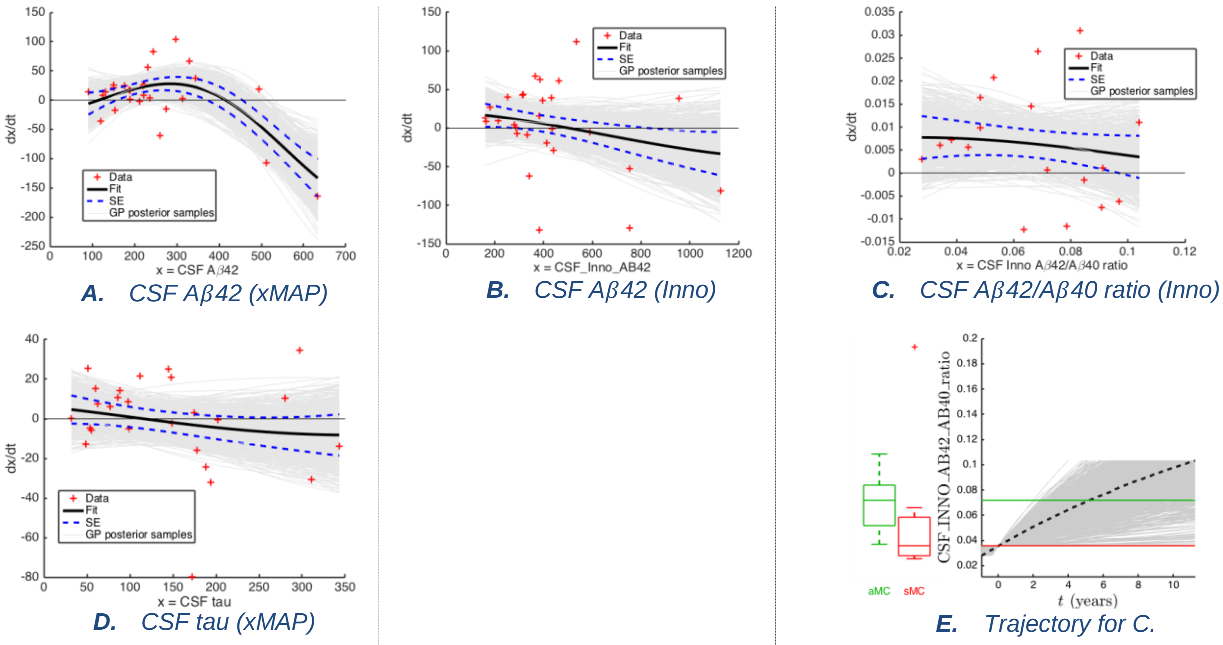

A. *CSF Aβ42 (xMAP)* ***B.*** *CSF Aβ42 (Inno)* ***C.*** *CSF Aβ42/Aβ40 ratio (Inno)*

D. *CSF tau (xMAP)* ***E.*** *Trajectory for C.*

*Supplementary Figure 14. Differential equation model fits for biomarkers where the approach could not infer a valid biomarker trajectory. In **A.**, **B.**, and **D.**, the non-monotonic dynamics precludes inference of a single trajectory. In **C.**, the fit implies an increasing biomarker (on average), but this biomarker is observed to decrease as disease progresses (Trajectory for **C.**).*

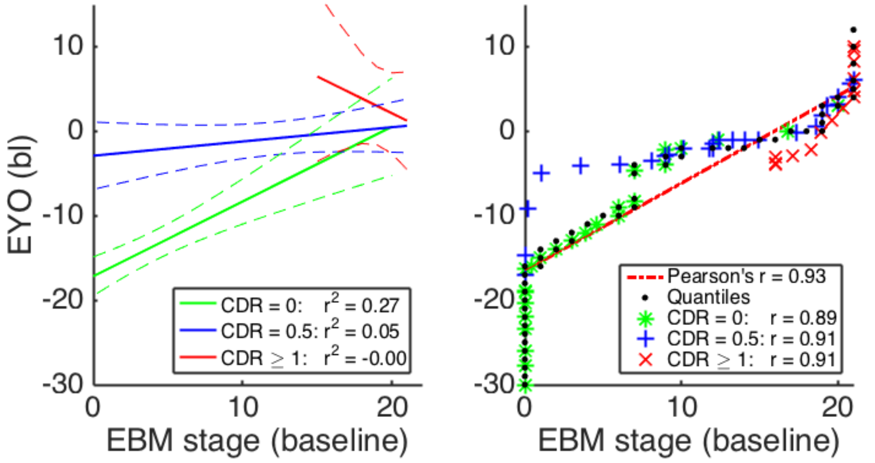

A. *Familial Estimated Years to Onset (EYO) vs Event-Based Model (EBM) stage.*

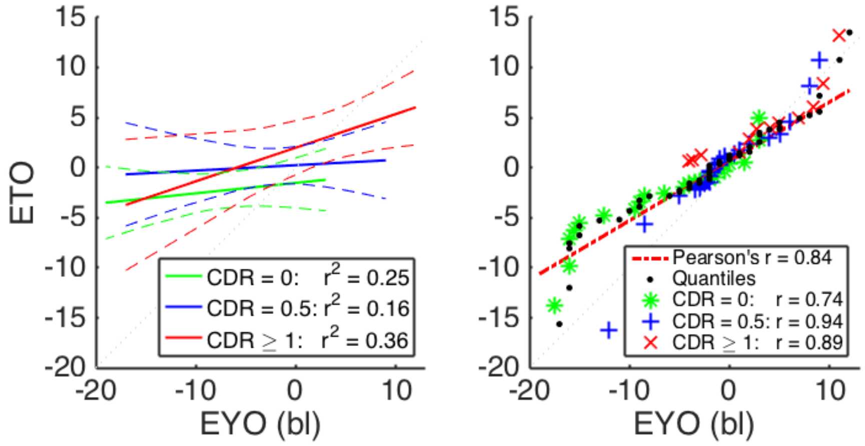

B. *Data-driven Estimated Time To Onset (ETO) vs familial estimated years to onset (EYO).*

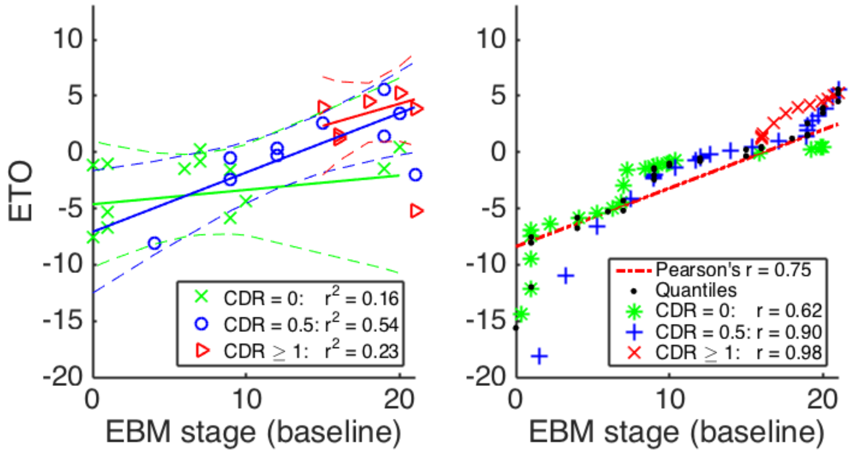

C. *Data-driven Estimated Time to Onset (ETO) vs Event-Based Model (EBM) stage.*

Supplementary Figure 15. Comparison of disease progression estimates for mutation carriers in the DIAN dataset. Clinical progression is defined using global Clinical Dementia Rating (CDR) score: asymptomatic (CDR = 0); mild symptomatic (CDR = 0.5); symptomatic (CDR > 0.5). Left panels: direct comparison of linear regression fits and 95% confidence intervals, with individual data points for EYO removed to avoid unblinding. Right panels: comparison of distributions via quantile plots, with a reference line (correlation) shown for all diagnostic categories combined. EYO = Estimated Years to Onset from parental age of onset; EBM = event-based model; ETO = Estimated Time to Onset from differential equation models; CDR = Clinical Dementia Rating; bl = baseline.

Supplementary Figure 15 shows correspondence between different estimates of disease progression: **A.** event-based model and familial EYO; **B.** differential equation model ETO and familial EYO; and **C.** event-based model and differential equation model ETO. In these figures, disease progresses from left to right and from bottom to top. Broadly speaking, Supplementary Figure 15 shows that all estimates of disease progression correctly predict unaffected/asymptomatic individuals (global Clinical Dementia Rating of 0) to lie towards the lower left corner and affected individuals (global Clinical Dementia Rating > 0) towards the upper right. Further, there is quite good linear correlation across all estimates, as shown by the linear regression fits in the left column, and by the quantile plots in the right column. In Supplementary Figure 15A, unaffected individuals should be found towards the lower left corner (EYO < 0; early event-based model stage), with affected individuals towards the top right corner (EYO > 0; late event-based model stage). Indeed, this is what we see, with good overall correlation between EYO and event-based model stage across diagnostic groups. For all groups of mutation carriers combined, a linear fit yields $R^2 \approx 0.45$. Supplementary Figure 15B shows a linear relationship between Estimated Time from Onset and familial EYO for all mutation carriers in the Dominantly Inherited Alzheimer Network dataset: both symptomatic and asymptomatic individuals. Supplementary Figure 15C compares our two modelling approaches by plotting differential-equation-model-based Estimated Time from Onset against event-based model stage.

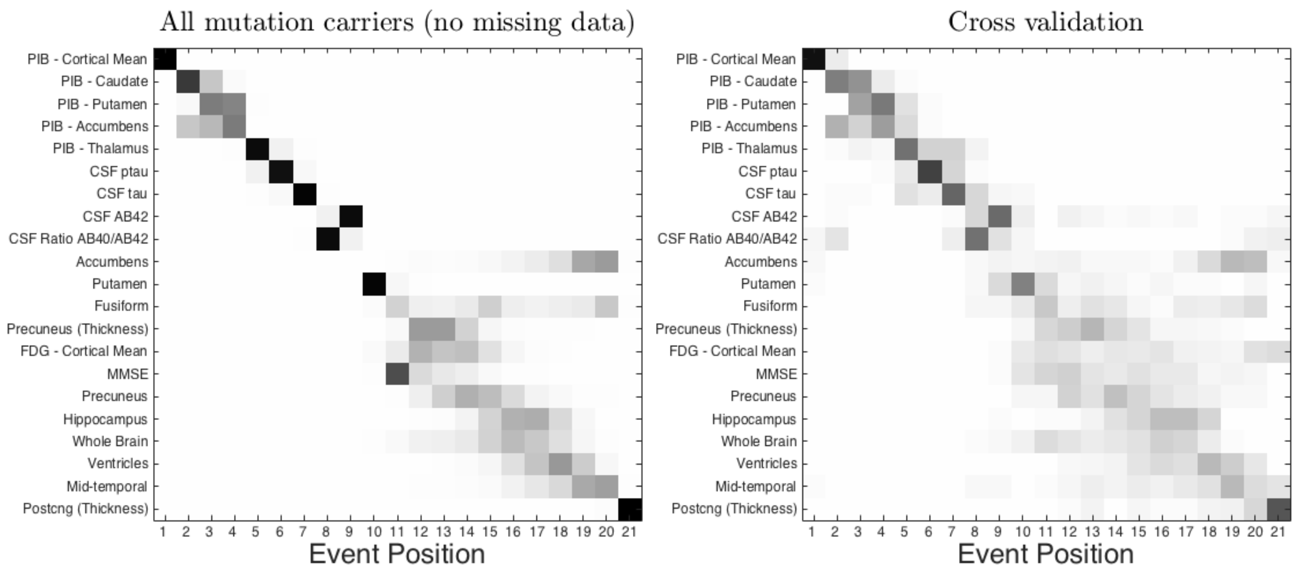

*Supplementary Figure 16. Event-based model of all mutation carriers in the Dominantly Inherited Alzheimer Network cohort, omitting participants with missing data. This result compares qualitatively very well with **Error! Reference source not found.**, which included participants with missing data imputed (see Statistical Analysis section). This supports the notion that our missing data imputation did not affect the estimated sequence of abnormality. Abbreviations as in **Error! Reference source not found.**.*

Biomarker	Smoothness	Scale	Residual
	Smoothness $\sqrt{1/\rho^2}$ $(\hat{R})$	Scale $\sqrt{\eta^2}$ $(\hat{R})$	Residual $\sqrt{\sigma^2}$ $(\hat{R})$
MMSE score	11.39 (1)	3.353 (1.001)	1.329 (1)
cross-validation	11.94 $\pm$ 1.26 (1$\pm$0)	3.379 $\pm$ 0.089 (1$\pm$0)	1.338 $\pm$ 0.053 (1$\pm$0)
CSF tau	393.3 (1)	29.06 (1)	23.54 (1)
cross-validation	390.9 $\pm$ 24.4 (1$\pm$0)	29.73 $\pm$ 2.50 (1$\pm$0)	23.45 $\pm$ 1.98 (1$\pm$0)
CSF p-tau	183.9 (1)	16.88 (1)	10.94 (1)
cross-validation	181.5 $\pm$ 9.8 (1$\pm$0)	17.33 $\pm$ 2.70 (1$\pm$0)	10.9 $\pm$ 0.6 (1$\pm$0)
CSF Aβ42 (xMAP)	426.4 (1)	145.3 (1)	39.91 (1)
cross-validation	445.1 $\pm$ 59.8 (1$\pm$0)	137.4 $\pm$ 20.5 (1$\pm$0)	40.09 $\pm$ 2.72 (1$\pm$0)
CSF Aβ42 (Inno)	1250 (1)	67.44 (1)	54.03 (1)
cross-validation	1240 $\pm$ 52 (1$\pm$0)	68.81 $\pm$ 6.45 (1$\pm$0)	53.56 $\pm$ 3.93 (1$\pm$0)
CSF Aβ42/Aβ40 ratio (Inno)	0.1173 (1)	0.01345 (1)	0.01226 (1)
cross-validation	0.1165 $\pm$ 0.0075 (1$\pm$0)	0.01409 $\pm$ 0.00097 (1$\pm$0)	0.01217 $\pm$ 0.00055 (1$\pm$0)
PiB – Caudate	1.205 (1)	0.4055 (1)	0.2882 (1)
cross-validation	3.774 $\pm$ 1.591 (1$\pm$0)	0.418 $\pm$ 0.065 (1$\pm$0)	0.2914 $\pm$ 0.0590 (1.001$\pm$0.001)
PiB – Putamen	5.747 (1)	0.3652 (1)	0.3223 (1)
cross-validation	5.662 $\pm$ 0.169 (1$\pm$0)	0.3793 $\pm$ 0.0177 (1$\pm$0)	0.3215 $\pm$ 0.0319 (1$\pm$0)
PiB – Nucleus Accumbens	5.307 (1)	0.5896 (1)	0.4522 (1)
cross-validation	5.29 $\pm$ 0.32 (1$\pm$0)	0.5916 $\pm$ 0.0481 (1$\pm$0)	0.4505 $\pm$ 0.0606 (1$\pm$0)
PiB – Cortical mean	6.585 (1)	0.4463 (1)	0.256 (1)
cross-validation	6.424 $\pm$ 0.132 (1$\pm$0)	0.4376 $\pm$ 0.0367 (1$\pm$0)	0.2553 $\pm$ 0.0157 (1$\pm$0.001)
PiB – Thalamus	3.542 (1.001)	0.2113 (1)	0.1951 (1)
cross-validation	3.486 $\pm$ 0.095 (1$\pm$0)	0.2181 $\pm$ 0.0072 (1$\pm$0)	0.1941 $\pm$ 0.0150 (1$\pm$0)
FDG – Posterior cingulate	1.971 (1)	0.1334 (1)	0.1056 (1)
cross-validation	1.93 $\pm$ 0.04 (1$\pm$0)	0.1349 $\pm$ 0.0054 (1$\pm$0)	0.1055 $\pm$ 0.0057 (1$\pm$0)
FDG – Hippocampus	1.082 (1)	0.05033 (1)	0.03827 (1)
cross-validation	1.065 $\pm$ 0.0192 (1$\pm$0)	0.05185 $\pm$ 0.00174 (1$\pm$0)	0.03819 $\pm$ 0.00189 (1$\pm$0)
FDG – Cortical mean	1.498 (1)	0.1367 (1)	0.09115 (1)
cross-validation	1.484 $\pm$ 0.051 (1$\pm$0)	0.1381 $\pm$ 0.0084 (1$\pm$0)	0.09091 $\pm$ 0.00627 (1$\pm$0)
Volume – Hippocampus	4747 (1)	781 (1)	344.3 (1)
cross-validation	5630 $\pm$ 1605 (1$\pm$0)	799.7 $\pm$ 44.6 (1$\pm$0)	346.2 $\pm$ 14.0 (1$\pm$0)
Volume – Mid-temporal gyrus	2.665×10^4 (1)	1626 (1)	787.7 (1)
cross-validation	$2.647\pm0.084\times10^4$ (1$\pm$0)	1625 $\pm$ 131 (1$\pm$0)	788.8 $\pm$ 46.9 (1$\pm$0)
Volume – Entorhinal cortex	5642 (1.001)	294.9 (1)	284.3 (1)
cross-validation	5621 $\pm$ 173 (1$\pm$0)	305.4 $\pm$ 12.4 (1$\pm$0)	283.9 $\pm$ 7.6 (1$\pm$0)

Volume – Fusiform gyrus	2.02 × 10^4 (1)	1752 (1)	723.7 (1)
cross-validation	2.03±0.09 × 10^4 (1±0)	1747 ± 109 (1±0)	725.5 ± 52.2 (1±0)
Volume – Caudate	7648 (1)	444.4 (1)	270 (1)
cross-validation	7423 ± 204 (1 ± 0)	471.7 ± 25.7 (1 ± 0)	269.1 ± 20.8 (1 ± 0)
Volume – Putamen	6027 (1)	1906 (1)	480.7 (1)
cross-validation	6561 ± 1139 (1 ± 0)	1849 ± 184 (1 ± 0)	483.3 ± 25.7 (1 ± 0)
Volume – Thalamus	1.442 × 10^4 (1)	779.5 (1)	548.7 (1)
cross-validation	1.428±0.029 × 10^4 (1 ± 0)	805.7 ± 20.6 (1 ± 0)	548.4 ± 21.4 (1 ± 0)
Volume – Nucleus accumbens	1597 (1)	127.5 (1)	99.95 (1)
cross-validation	1586 ± 31 (1 ± 0)	130.7 ± 4.1 (1 ± 0)	99.99 ± 6.28 (1 ± 0)
Volume – Precuneus	1.966 × 10^4 (1)	1515 (1)	657.3 (1)
cross-validation	2.015±0.117 × 10^4 (1 0)	1509 ± 107 (1 ± 0)	658.5 ± 30.7 (1 ± 0)
Cortical thickness – Posterior cingulate	2.009 (1)	0.1004 (1)	0.06954 (1)
cross-validation	2.004 ± 0.078 (1 ± 0)	0.1017 ± 0.0070 (1 ± 0)	0.06934 ± 0.00319 (1 ± 0)
Cortical thickness – Precuneus	1.912 (1)	0.1466 (1)	0.0577 (1)
cross-validation	1.908 ± 0.056 (1 ± 0)	0.1478 ± 0.0073 (1 ± 0)	0.0509 ± 0.0029 (1 ± 0)
Cortical thickness – Entorhinal	3.647 (1)	0.3463 (1)	0.1937 (1)
cross-validation	3.583 ± 0.187 (1 ± 0)	0.3496 ± 0.0265 (1 ± 0)	0.1936 ± 0.0133 (1 ± 0)
Cortical thickness – Fusiform gyrus	2.344 (1)	0.1583 (1)	0.06472 (1)
cross-validation	2.354 ± 0.121 (1 ± 0)	0.1548 ± 0.0147 (1 ± 0)	0.06472 ± 0.00388 (1 ± 0)
Cortical thickness – Mid-temporal gyrus	1.947 (1)	0.1864 (1)	0.07265 (1)
cross-validation	1.965 ± 0.070 (1 ± 0)	0.1827 ± 0.0048 (1 ± 0)	0.07295 ± 0.00361 (1 ± 0)
Volume – Ventricles	9.091 × 10^4 (1)	1.625 × 10^4 (1)	2625 (1)
cross-validation	8.998±0.664 × 10^4 (1 ± 0)	1.623±0.591 × 10^4 (1±0)	2624 ± 161 (1 ± 0)
Volume – Whole brain	5.86 × 10^5 (1)	5.783 × 10^4 (1)	1.294 × 10^4 (1)
cross-validation	5.812±0.3808 × 10^5 (1 ± 0)	5.708±0.231 × 10^4 (1±0)	1.297±0.641 × 10^4 (1 ± 0)

Supplementary Table 1. Differential equation model regression results: Gaussian process hyperparameter estimates. Units – CSF: pg/mL (except ratio); PET (PiB and FDG): standardized uptake value ratio (SUVR) relative to the cerebellum; Volumes: mm^3; Cortical Thickness: mm. Ten-fold cross validation shows mean ± standard deviation. The potential scale reduction factor $\hat{R}$, here given precise to three decimal places, indicates convergence of the algorithm: values close to 1 indicate strong convergence.
